# Type II-Metacaspases are involved in cell stress but not in cell death in the unicellular green alga *Dunaliella tertiolecta*

**DOI:** 10.15698/mic2019.11.696

**Published:** 2019-10-07

**Authors:** M. Teresa Mata, Armando Palma, Candela García-Gómez, María López-Parages, Víctor Vázquez, Iván Cheng-Sánchez, Francisco Sarabia, Félix López-Figueroa, Carlos Jiménez, María Segovia

**Affiliations:** 1 Department of Ecology, Faculty of Sciences, University of Málaga, Blvd. Louis Pasteur s / n, 29071-Málaga, Spain.; 2 Department of Organic Chemistry, Faculty of Sciences, University of Málaga, Blvd. Louis Pasteur s / n, 29071-Málaga, Spain.; & Present address: Antofagasta Bioinnovation Center (CBIA), Department of Biotechnology, Faculty of Marine Sciences and Biological Resources. University of Antofagasta, Antofagasta, Chile.; # Present address: Spanish Oceanographic Institute, Puerto Pesquero, 29640-Fuengirola, Málaga, Spain.

**Keywords:** Dunaliella tertiolecta, ultraviolet radiation, cell death, cell viability, metacaspases, caspase-like proteases, environmental stress

## Abstract

Ultraviolet radiation (UVR; 280–400 nm) has a great impact on aquatic ecosystems by affecting ecophysiological and biogeochemical processes as a consequence of the global change scenario generated by anthropogenic activities. We studied the effect of PAR (P)+UVA (A)+UVB (B) i.e. PAB, on the molecular physiology of the unicellular green alga *Dunaliella tertiolecta* for six days. We assessed the relationship between the triggered UVR stress response and metacaspases and caspase-like (CL)activities, which are proteases denoted to participate in cell death (CD) in phytoplankton. UVR inhibited cell growth and *in vivo* chlorophyll a fluorescence but did not cause cell death. Western blot analyses reflected that Type-II metacaspases (MCs) are present and appear to be involved in UVR induced-cell stress but not in dark-induced CD in *D. tertiolecta*. Enzyme kinetics revealed that cleavage of the MCs-reporter substrates RVRR, QRR, GRR, LKR, HEK, and VLK was 10-fold higher than WEHD, DEVD, IETD, and LETD CLs-substrates. The lowest apparent Michaelis-Menten constants (K_M_^ap^) corresponded to RVRRase (37.5 μM) indicating a high affinity by the RVRR substrate. The inhibition of enzymatic activities by using inhibitors with different target sites for hydrolyses demonstrated that from all of the R/ Kase activities only RVRRase was a potential candidate for being a metacaspase. In parallel, zymograms and peptide-mass fingerprinting analyses revealed the identities of such Rase activities suggesting an indirect evidence of possible natural physiological substrates of MCs. We present evidence of type II-MCs not being involved in CD in *D. tertiolecta*, but rather in survival strategies under the stressful irradiance conditions applied in this study.

## INTRODUCTION

The term “global change” encompasses planetary scale changes to atmospheric and ocean circulation, climate, element cycles, sea-ice and sea-level changes, food webs, biological diversity, pollution, health and fish stocks. Global change also affects exposure of organisms to solar ultraviolet B (UVB, 280–320 nm), ultraviolet A (UVA, 320–400 nm) and photosynthetically active radiation (PAR, 400-700 nm) through variations in the stratospheric ozone concentration, aerosol content and cloud cover [1–3]. Stratospheric ozone loss due to anthropogenic emission of chlorofluorocarbons (CFCs) raised the UVB doses reaching the Earth's surface owing to the ozone hole discovered in Antarctica in the mid 80's [1, 4]. However, even after the implementation of the Montreal Protocol to curtail CFCs emissions, ultraviolet radiation (UVR) has increased during stratospheric low-ozone events in northern latitudes [5–7]. The level of ozone depletion (accounting for ~ 6% UVB gain in the Northern Hemisphere) persists into the 21^st^ century caused by the long time required for ozone recovery [1, 8]. UVR is therefore still considered a global change stressor since rapid Arctic ozone losses are predicted to occur in the future. Additionally, climate change is provoking the cooling of the stratosphere favouring ozone degradation [9, 10].

The biological effective energy per photon in the UVR wavelengths is high and it affects numerous physiological, photobiological and photochemical processes. While UVB produces deleterious effects on both terrestrial and aquatic systems, many of the effects of solar radiation are caused by wavelengths corresponding to the UVA range as well, which is not influenced by fluctuations in the stratospheric ozone [11]. Marine phytoplankton contributes to half of the world's total primary production, accounting for ca. 50 % of the global atmospheric CO_2_ sequestration [12]. UVR has negative effects on phytoplankton 's physiology, including the inhibition of nutrient uptake, DNA and antennae damage, and decreased carbon assimilation mechanisms [11]. This decisively affects primary production in phytoplankton [13], driving microalgae into decreased cell viability and, in most species, leading to cell death in both cultures and natural communities [14–19].

The occurrence of programmed cell death (PCD) as an intrinsically instituted cellular process by which cell dismissal takes place as a consequence of biotic and/or abiotic stress has been widely reported in phytoplankton (reviewed by [20]). PCD in unicellular microalgae is justified in terms of the population level [21] or species-specific fitness effects at the community level [22] given that apparently cell death would not confer any obvious evolutionary advantage to unicellular organisms. However, most of the studies seem to disregard that the “execution” of PCD in non-metazoan organisms is significantly different from PCD in metazoans considering morphological, enzymological and functional aspects, thereby lacking a number of key molecular components of the metazoan PCD machinery [23]. The evolution of PCD in microorganisms and the accompanying terminology is beyond the scope of this paper. However, in light of the number of discrepancies concerning the interpretation of the term PCD and the actual heated-debate about revisiting the PCD nomenclature in unicellular organisms [23–25] we consider the cell death interpretation by [26] as the appropriate one for phytoplankton. Accordingly, we will simply refer to intrinsic (non-accidental) forms of mortality as “cell death” (CD) vs. “accidental cell death” (ACD). The question arises hereby on the enzymes involved in CD in phytoplankton and which is their hydrolytic nature.

Both caspase-like proteases (CLs) and metacaspases (MCs) have been appointed to participate in CD in phytoplankton (reviewed by [20]). Dunaliella tertiolecta cultures subjected to continuous darkness showcased massive CD and CLs measured in parallel matched the sequence of the death event [27]. Interestingly, MCs participation in the response to stress or CD has not been studied in *D. tertiolecta* up to now, and it still remains largely unexplored in other organisms as well. MCs are members of the clan CD of cysteine proteases, presenting a caspase-hemoglobinase fold that encloses a conserved cysteine–histidine catalytic dyad. MCs are found in fungi, plants and protists (unicellular eukaryotes) and also participate in CD (reviewed by [28] and [29]). These enzymes are distinct from caspases and caspase-like proteases in terms of target site specificity. Their target substrate sites contain either arginine (R) or lysine (K) at the P1 position, different to that of caspases or caspase-like proteases that cleave after aspartate (D) in P1 [30]. In fact, the most relevant biochemical feature of all MCs is the strict R and K substrate specificity, which distinguishes them from caspases [28, 31, 32]). Following this argument, what has been measured before in phytoplankton with caspase-specific substrates as “MCs activities” is not, in fact, due to MCs, because MCs do not possess caspase or caspase-like proteolytic activity [33–36]. Moreover, subtilases (SBT) from the serine protease family proteins, perform CLs hydrolysis in plants [37] after aspartate residues as for example phytaspases (aspartate-specific proteases, [38, 39]), or the vacuolar processing enzyme [40]. Strikingly, CD was not detected in the unicellular green alga D. tertiolecta stressed with UVR in repeated experiments of this study. Cells survived chronic UVR exposure by induction of DNA repair mechanisms [41] in which the activation of antioxidant enzymes had a priority role scavenging reactive oxygen species (ROS) [18], alternative photoprotective mechanisms were triggered [42] and repair-genes were actively transcribed [43]. CLs were measured in these studies despite of cells not being dead, therefore suggesting an underlying UVR-managing stress role for CLs.

The aim of the present work was to study whether MCs were involved in the cellular stress response to chronic UVR exposure in the marine unicellular green alga D. tertiolecta (Viridiplantae) and the meaning of it by (1) MCs immunodetection and accumulation pattern, (2) characterising the potential MCs enzymatic activities by kinetic analysis, (3) studying enzymatic activity inhibition kinetics, (4) zymograms and peptide-mass-fingerprinting analyses to ascribe protein identities to detected proteins and, (5) to differentiate between CL activities and MCs in this species resilience to CD under UVR stress. Microalgae from the genus Dunaliella are well known for their extraordinarily high tolerance to abiotic stress [44]. D. tertiolecta was originally isolated from a Norwegian fjord close to the Arctic Circle. The UVR ratio and continuous treatment in the present experimental approach was selected as an extreme condition to simulate the long UVR exposure periods observed at high latitudes (key planetary locations for global change-related impact surveillance) during summer and future predicted conditions [9]. Such features make these microalgae an appropriate biological model for studying environmental stress responses.

## RESULTS

### Ultraviolet radiation inhibits cell growth and chlorophyll a fluorescence emission

**Figure 1:**
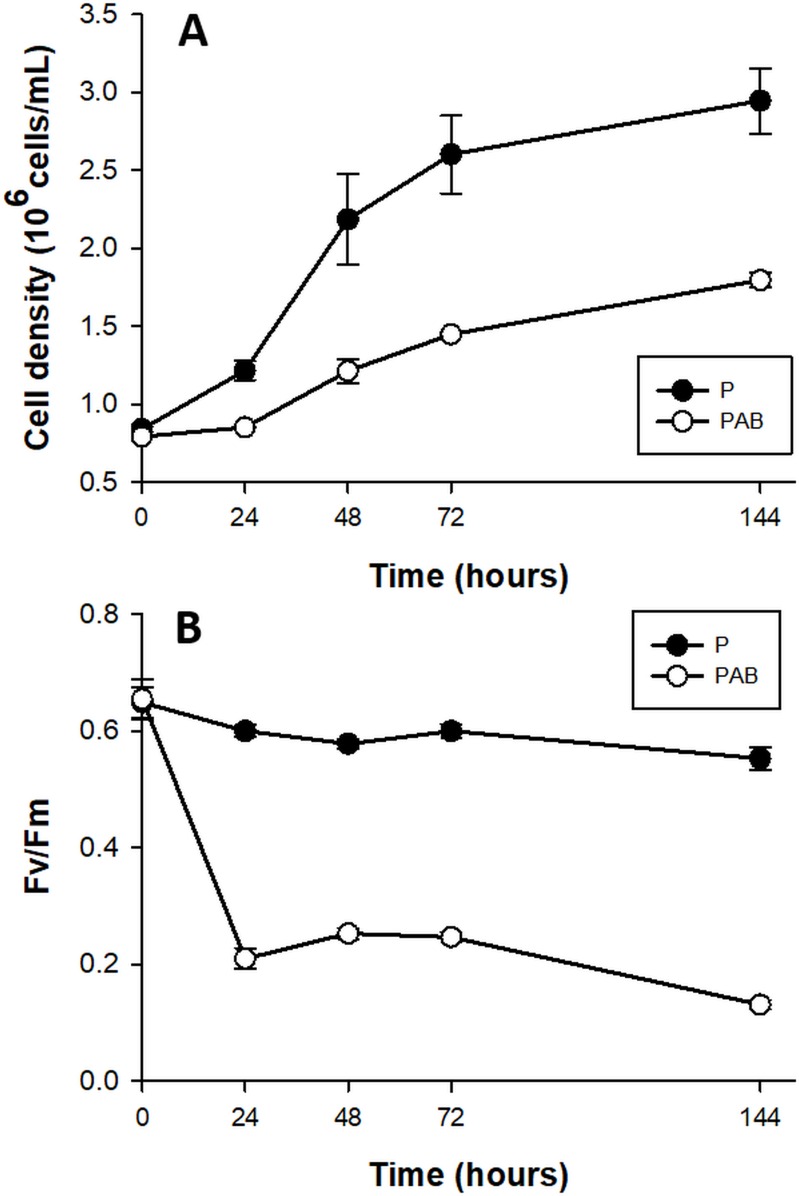
FIGURE 1. Cell abundance **(A)** and maximal quantum yield (F_v_/F_m_) **(B)** in cultures of *D. tertiolecta* exposed to PAR (P, •) and to PAR + UVA + UVB (PAB, ○) for six days immediately after the day 0 measurement (culture in P). Symbols are means of measurements of two independent replicate cultures and three replicate samples from each culture cylinder. Error bars indicate standard deviations. Figure reproduced with permission of The Journal of Experimental Botany [41].

The growth rates of D. tertiolecta cells **(Fig. 1A)** decreased 2-fold in PAB (μ_PAB_ = 0.58 day^-1^) compared to PAR (μP = 1.01 day^-1^) (p < 0.05) (P treatment published in [41] and included in the plot for comparison). F_v_/F_m_ **(Fig. 1B)** acutely dropped off during the first 24 h by 78% under PAB in contrast to PAR, where F_v_/F_m_ was within the typical range for healthy cells (0.65) (F_v_/F_m_ values reported in [41] are included for comparison).

### Type-II MCs are involved in UVR induced-cell stress but not in CD

**Figure 2:**
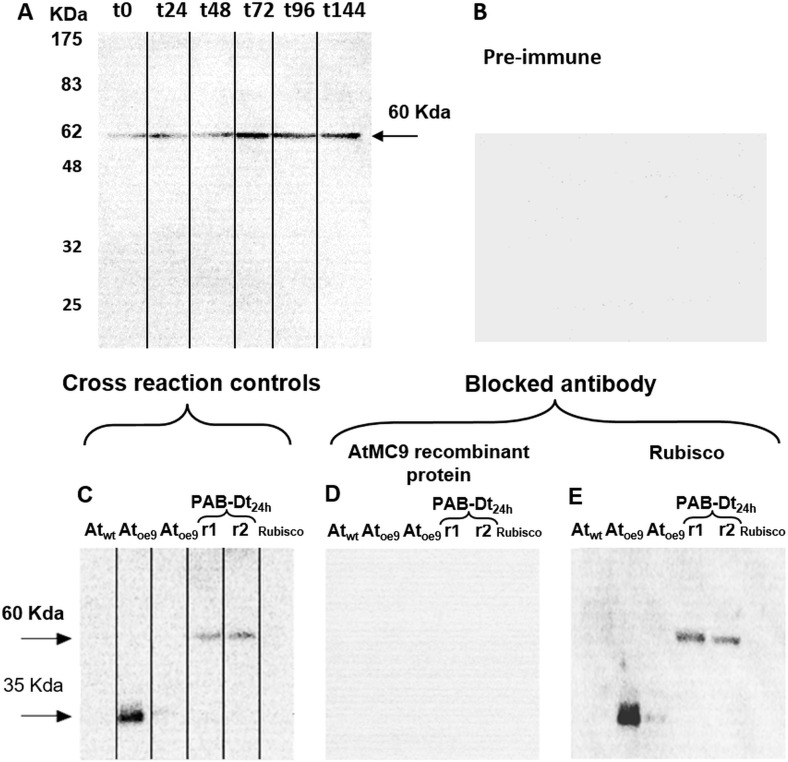
FIGURE 2. MCs immunodetection in *D. tertiolecta* exposed to PAR + UVA + UVB (PAB) for six days with a polyclonal antibody specific for MC9 (α-AtMC9) from the model plant *A. thaliana* **(A)**; negative control with the 1^ary^ antibody substituted by its correspondent pre-immune sera **(B)**; positive cross-reaction controls consisting of proteins extracted from *A. thaliana* wild type leaves (Atwt), from *A. thaliana* over-expressing MC9 (Atoe9) and MC1 ( Atoe1 ) electrophoresed and western blotted together with *D. tertiolecta* samples (Dt r1 and r2) and commercial pure rubisco protein probed against α-AtMC9 **(C)**; positive control consisting of α-AtMC9 blocked with MC9 recombinant protein (RCMC9) and probed against Atwt, Atoe9, Atoe1, Dt r1 and r2 and pure rubisco protein **(D)**; negative control consisting of α-AtMC9 blocked with pure rubisco and probed against Atwt, Atoe9, Atoe1, Dt r1 and r2 and pure rubisco **(E)**.

Immunodetection demonstrated the presence of Type-II MCs in D. tertiolecta. Western blots probed with the specific antibody against MC9 (α-AtMC9) revealed one unique band of increasing intensity from t0 to t144 corresponding to 60 KDa **(Fig 2A)** at high antibody dilution, hence demonstrating elevated specificity. The membranes probed with the pre-immune sera as control were blank **(Fig 2B)**. Controls consisting of membranes containing proteins extracted from the *Arabidopsis thaliana* sub-type over-expressing MC9 (Atoe9) and with *D. tertiolecta* extracted proteins at 48h-PAB crossed-reacted with α-AtMC9. Two unique bands of 35 and 60 KDa respectively were detected **(Fig 2C)**. However, no bands appeared with α-AtMC9 in blots from proteins extracted from *A. thaliana* wild type (Atwt) leaves, and the *A. thaliana* sub-type over-expressing MC1 (Atoe1), nor with pure rubisco **(Fig. 2C)**. The next control consisted of α-AtMC9 blocked with the MC9 recombinant protein (RCMC9). It did not cross-react at all with any extracted proteins from *D. tertiolecta* and *A. thaliana* or with rubisco **(Fig. 2D)**. The last control corresponded to α-AtMC9 blocked with pure rubisco. In this case, the 60 KDa band from *D. tertiolecta* and the 35 KDa band from *A. thaliana* were again detected **(Fig. 2E)**.

**Figure 3:**
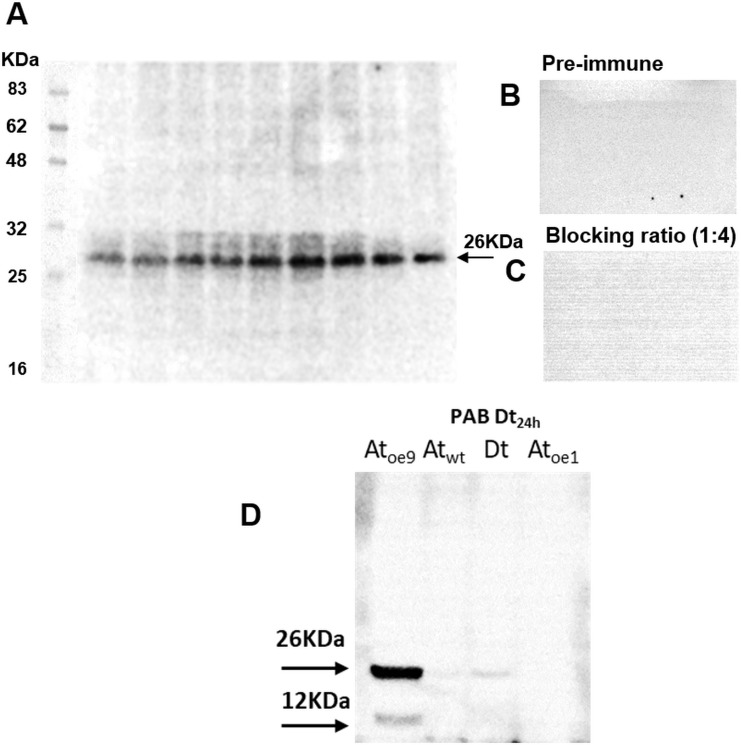
FIGURE 3. MCs immunodetection in *D. tertiolecta* exposed to PAR + UVA + UVB (PAB) for six days with a polyclonal antibody specific against MC-type II (α-AtII) from the model plant *A. thaliana* **(A)**; negative control with the 1^ary^ antibody substituted by its correspondent pre-immune sera **(B)**; positive control consisting of α-AtII blocked with MC9 recombinant protein (RCMC9) and probed against Atwt, Atoe9, Atoe1, and Dt **(C)**; positive cross-reaction controls consisting of proteins extracted from *A. thaliana* wild type leaves (Atwt), from *A. thaliana* over-expressing MC9 (Atoe9) and MC1 (Atoe1) electrophoresed and western blotted together with *D. tertiolecta* samples (Dt) protein and probed against α-AtII **(D)**.

**Figure 4:**
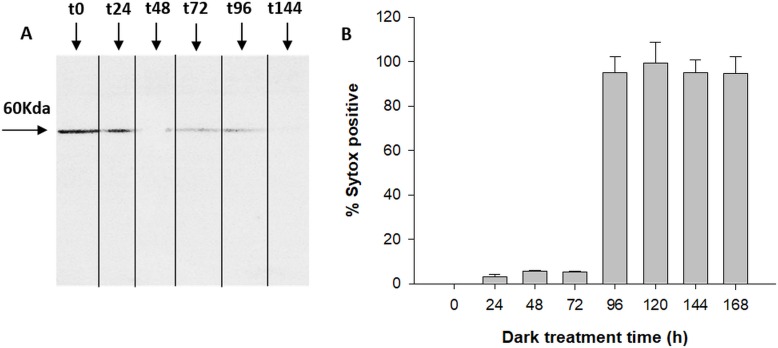
FIGURE 4. MCs immunodetection in *D. tertiolecta* during dark-induced CD **(A)**; Percentage of dead cells based on Sytox-green staining for seven days dark-induced CD in *D. tertiolecta* cultures **(B)**. Symbols are means of measurements of two independent replicate cultures and three replicate samples from each culture cylinder. Error bars indicate standard deviations.

The antibody specific against type II-MCs (α-AtII) crossed-reacted with proteins of *D. tertiolecta* showing a 26 KDa band of increasing intensity over the experimental time **(Fig. 3A)** and many other less intense bands. The membranes probed with both the α-AtII blocked with RCMC9 and with the pre-immune sera as controls were blank **(Fig 3B, C)**. The control consisting of proteins from Atoe9 crossed-reacted with α-AtMC9 and two bands of ≈26 KDa and 12 KDa were observed. Proteins from Atwt and *D. tertiolecta* at 48h-PAB were specifically recognised by α-AtMC9, showing one band of ≈26 kDa. In contrast, proteins from Atoe1 were not recognized **(Fig. 3D)**. The antibody against type I-MCs (α-AtI) crossed-reacted with the pre-immune sera, therefore indicating its lack of specificity and so we avoided the use of such antibody in subsequent detections (data not shown). Dark-treated *D. tertiolecta* samples were used as a positive control for CD measured with the CD fluorescent probe Sytox-green and probed with α-AtMC9 to check for MCs involvement in *D. tertiolecta* cell demise. The 60 KDa band accumulation pattern and positive-Sytox-green labelled dead cells proved to be inverse **(Fig 4 A, B)**.

### The cleavage rate of MCs-reporter substrates is 10-fold higher than CLs-substrates

**Figure 5:**
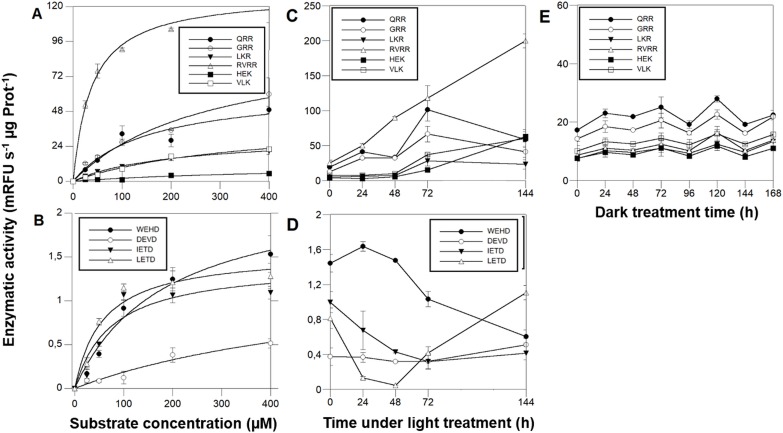
FIGURE 5. MCs and CL enzymatic activities in *D. tertiolecta* exposed to PAR + UVA + UVB (PAB) for six days. Michaelis-Menten kinetics showed by Boc-7-amino-4-fluoromethyl coumarin (AMC) reporters of the substrates RVRR (Δ), QRR (•), GRR (○), LKR (▾), HEK (▪), and VLK (□) **(A)**; Michaelis-Menten kinetics showed by Ac-7-amino-4-trifluoromethyl coumarin (AFC) reporters of the substrates WEHD (•), DEVD (○), IETD (▾), and LEHD (Δ) **(B)**; Cleavage activity showed by *D. tertiolecta* under PAB of (AMC) reporters of the substrates RVRR (Δ), QRR (•), GRR (○), LKR (▾), HEK (▪), and VLK (□) **(C)**; Cleavage activity showed by *D. tertiolecta* under PAB of (AFC) reporters of the substrates WEHD (•), DEVD (○), IETD (▾), and LETD (Δ) **(D)**; Cleavage activity showed by *D. tertiolecta* during dark-induced CD of (AMC) reporters of the substrates with R in P1 **(E)**, symbols as in **(A)**. Symbols are means of measurements of two independent replicate cultures and three replicate samples from each culture cylinder. Error bars indicate standard deviations.

MCs activities were ≈ 2 orders of magnitude higher than CL enzymatic activities. Michaelis-Menten kinetics **(Fig. 5A, B, Table 1)** showed that among the MCs reporter substrates, RVRR with K_M_^ap^=37.50 μM **(Fig. 5A)** induced the highest significant enzymatic affinity, around 10 to 5-fold higher than the other MCs substrates **(Table 1)**. All of the CLs reporter substrates showed a K_M_^ap^ between 58-100 μM **(Fig. 5B)** except DEVD with ca. 6-fold less affinity. Protease activities varied during the time-course under PAB. The most remarkable result was the RVRRase behaviour **(Fig. 5C)** which was different from the rest of the potential MCs substrates. It increased exponentially up to 10-fold over time (p<0.01). All of the rest of the substrates containing R in P1 showed a significant (p<0.05) increase at 72h that decreased by the end of the experiment **(Fig. 5C)**. The substrates containing K in P1 raised steadily over time (p<0.05) **(Fig. 5C)**. Rase enzymatic activity in dark-induced CD samples showed a bi-phasic pattern by increasing and decreasing alternatively but there were no significant increases (p>0.05) of activity along the time course **(Fig. 5E)**.

**TABLE 1.** 

**AMC/AFC-Reporter substrate**	**Aminoacidic sequence**	**K_M_^ap^ (μM)**
RVRR-AMC	Ac-Arg-Val-Arg-Arg-AMC	37.504
QRR-AMC	Ac-Gln-Arg-Arg-AMC	153.33
LKR-AMC	Ac-Leu-Lys-Arg-AMC	196.09
GRR-AMC	Ac-Gly-Arg-Arg-AMC	281.7
VLK-AMC	Ac-Val-Leu-Lys-AMC	337.62
HEK-AMC	Ac-His-Glu-Lys-AMC	452.63
IETD-AFC	Ac-Ile-Glu-Thr-Asp-AFC	57.48
LETD-AFC	AC-Leu-Glu-Thr-Asp-AFC	75.28
VEID-AFC	Ac-Val-Glu-Ile-Asp-AFC	100.00
WEHD-AFC	Ac-Trp-Glu-His-Asp-AFC	186.10
DEVD-AFC	Ac-Asp-Glu-Val-Asp-AFC	637.67

Specific aminoacidic sequences of the Boc-7-amino-4-fluoromethyl coumarin (AMC) reporters of the substrates QRR, GRR, LKR, RVRR, HEK, and VLK used to detect potential MCs; Ac-7-amino-4-trifluoromethyl coumarin (AFC) reporters of the substrates WEHD, DEVD, VEID, IETD, and LEHD assayed to detect CL activities; K_M_^ap^ is the apparent Michaelis-Menten constant obtained after incubation of cell lysates with increasing concentrations (μM) of each reporter substrates.

IETDase, DEVDase and WEHDase activities declined during the first 24-48h to recover by the end of the experiment (p<0.05). LETDase activity did not change **(Fig. 5D)**.

### RVRRase is a potential candidate for being a MC

**Figure 6:**
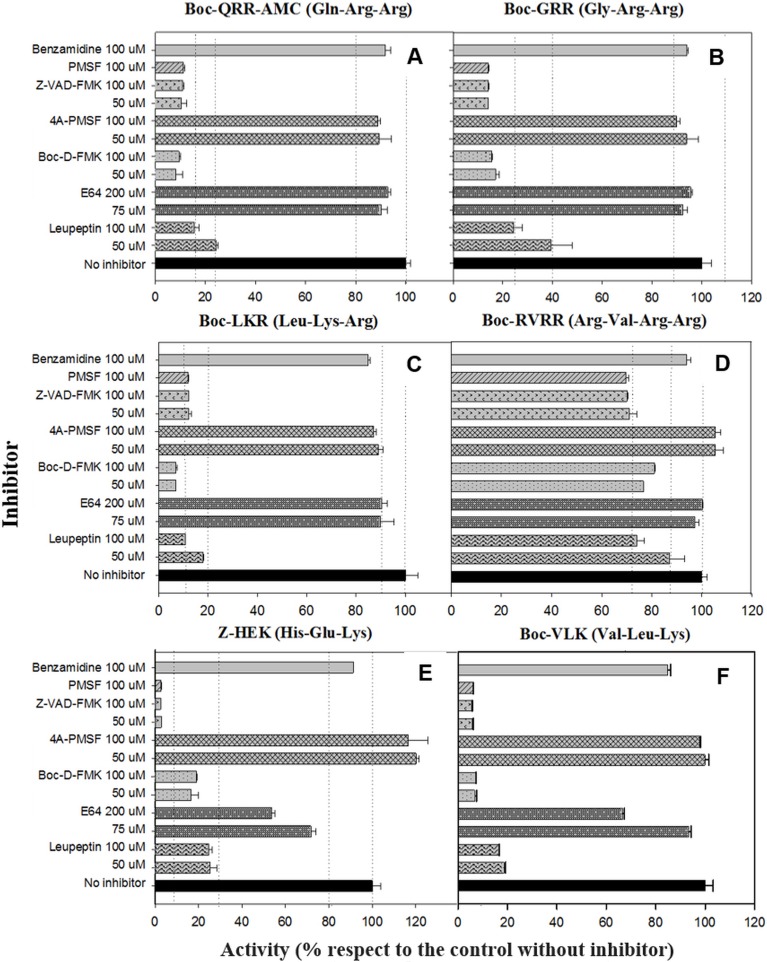
FIGURE 6. Inhibition of Rase and Kase enzymatic activities with several reversible and irreversible inhibitors at different concentrations. RVRRase **(A)**; GRRase **(B)**; QRRase **(C)**; LKRase **(D)**; VLKase **(E)**; HEKase **(F)**.

**Figure 6** depicts the inhibition of the MC reporter substrates cleavage by different inhibitors and concentrations. All of the enzymatic activities with R or K in P1, except RVRRase **(Fig. 6A)** suffered a significant inhibition by nearly all the inhibitors used. The reversible serine proteases inhibitor PMSF prevented the enzymatic activity by 90% (p<0.01). However, enzymatic activities were not inhibited (p>0.05) by the irreversible inhibitor form 4A-PMSF. The irreversible metazoan-caspase inhibitors Z-VAD-FMK and BOC-D-FMK also inhibited the activities by ca. 90 % (p<0.05). In contrast, the irreversible cysteinyl-proteases inhibitor E64 did not inhibit Rase activities significantly (p>0.05) **(Fig.6B–D)**, but Kase activities declined 30% (average) **(Fig.6E, F)**. Leupeptin, a reversible serine and cysteinyl-proteases inhibitor, diminished all the enzymatic activities by ≈ 80 % except for RVRRase that only dropped off by 15 % (average) (p<0.05). The poly-ADPribose polymerase (PARP) inhibitor benzamidine, slightly depleted all the activities around 10 % (p<0.05).

**Figure 7:**
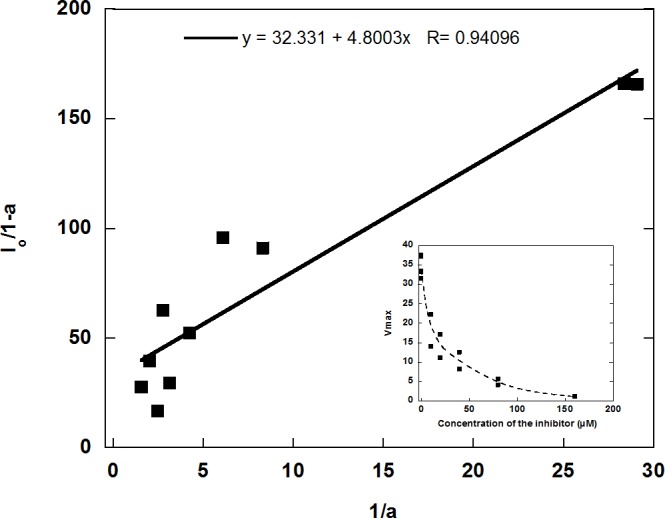
FIGURE 7. Inhibition kinetics of RVRRase activity with the Ac/Dec-RVRR-CMK irreversible inhibitor, in which a is the ratio between Vmax in the presence of the inhibitor (Vmax_i_) and Vmax in the controls in the absence of the inhibitor (Vmax_0_) (a= Vmax_i_/Vmax_0_), and I_o_ is each of the concentrations of inhibitor tested in the assays.

In view of the lack of inhibition, RVRRase was a potential candidate for being a MC. The specific irreversible inhibitor Ac/Dec-RVRR-CMK was then tested at different concentrations as specified in the methodology section. It inhibited RVRRase activity following a logarithmic trend **(Figure 7)** and the K_i_^ap^ occurred at 5.19 μM. Therefore, we can assume that 90-95 % inhibition took place around 29.5 μM.

### Zymograms and peptide-mass fingerprinting analyses revealed the identities of the Rase activities

**Figure 8:**
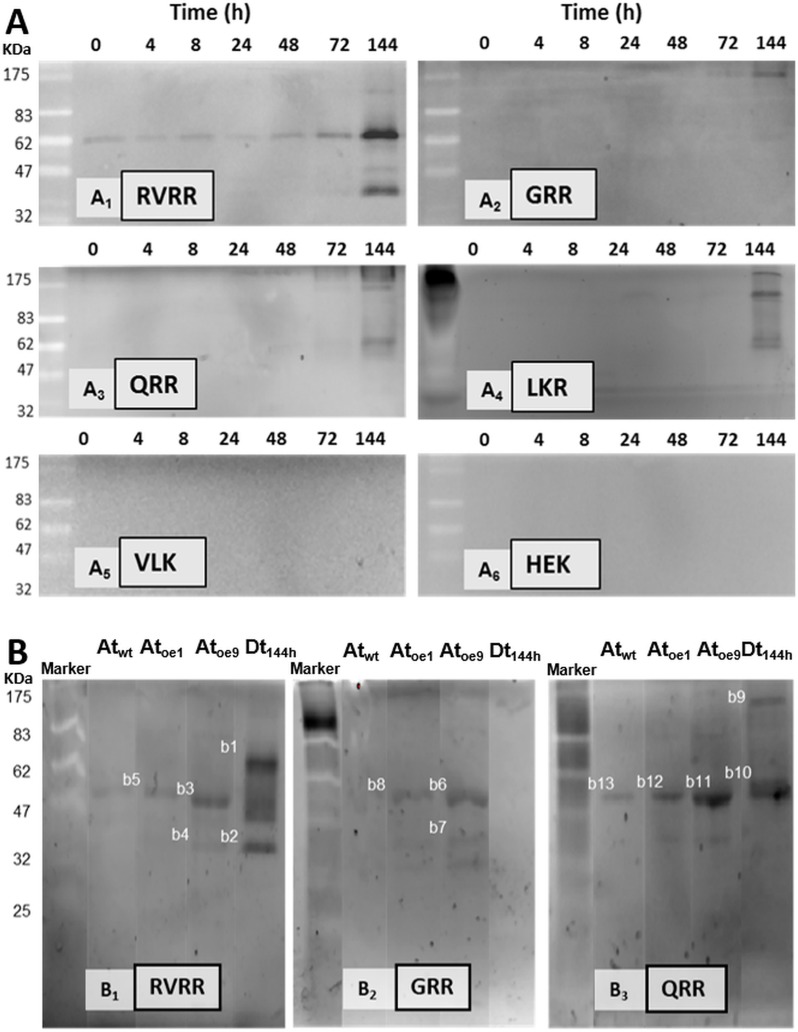
FIGURE 8. Zymograms of Rase and Kase activities in *D. tertiolecta* exposed to PAR + UVA + UVB (PAB) for six days. RVRRase **(A1)**; GRRase **(A2)**; QRRase **(A3)**; LKRase **(A4)**; VLKase **(A5)**; HEK **(A6)**. Controls consisting of zymograms of proteins extracted from *A. thaliana* wild type leaves (Atwt), from *A. thaliana* over-expressing MC9 (Atoe9) and MC1 (Atoe1) electrophoresed and western blotted together with *D. tertiolecta* samples (Dt) exposed to PAB for 144 h (six days).

Five bands of ≈ 100 KDa, 60 KDa, 50 KDa, 40KDa and 26KDa exhibited RVRRase activity in zymograms **(Fig. 8A1)**. The 60 KDa was the clearest band detected following an accumulation pattern over the experimental time that peaked at 144 h. In GRR **(Fig. 8A2)** and QRR **(Fig. 8A3)** zymograms only the 100 KDa and 60 KDa bands could be clearly seen. In LKR **(Fig. 8A4)** zymograms bands were never well resolved, an so we had to discard it for electroelution purposes. VLK and HEK zymograms were blank **(Fig. 8A5, 6)**. Control zymograms consisting of RVRRase **(Fig. 8B1**, bands B1 to B5), GRRase **(Fig. 8B2**, bands B6 to B8) and QRRase activities **(Fig. 8B3**, bands B9 to B13) from *D. tertiolecta* at 144 h-PAB, Atwt, Atoe9 and Atoe1, showed the bands described above and they were also excised for electroelution. The identity of the bands obtained in the zymograms after MaldiTOF/TOF analyses are summarised in Table 2S-suplemental material. The highest significant similarity from bands with RVRRase activity from *D. tertiolecta* were B1 and B2. Band B1 (60KDa) presented similarity with Phytochrome B from *Cleome hassleriana*, myb family transcription factor from *A. thaliana* and a 110 KDa 4SNc-Tudor domain protein from *Pisum sativum*, all of them Embryophytes. The analysis also revealed similarity of such band with predicted proteins from other viridiplantae. Band B2 (≈ 40KDa) showed significant similarity with predicted proteins from both the Bryophyte *Physcomitrella patens* and the Embryophyte *Vitis vinífera*. GRRase in-gel from *D. tertiolecta* did not display any activity **(Fig. 8B2**, lane Dt_144h_). Band B10 (60KDa) with QRRase activity showed again significant similarity with predicted proteins from *P. patens* and *V. vinífera*. However, B9 (≈ 100 KDa) revealed similarity with rubisco large subunit from different Brassicaceae. Sixty-KDa bands from *A. thaliana* presented similarity among the different Rase substrates. B5 and B13 from Atwt; B3, B8 and B12 from Atoe1 and B11 from Atoe9 significantly matched with rubisco large subunit from several Embryophytes. Band B7 highly scored with a putative glyceraldehyde-3-phosphate dehydrogenase from *A. thaliana*.

## DISCUSSION

UVR at the stressful doses used in this experiment (chronic exposure with a high UVR: PAR ratio) did not induce CD in the unicellular green alga D. tertiolecta [18, 41–43]. Despite that cells showed signs of stress such as inhibition of cell growth and decreased chlorophyll fluorescence emission **(Fig. 1)**, they survived. The UVR tolerance-response has been shown to be dose-dependent, and repair under UVR is a key element in *D. tertiolecta* resilience [18, 41–43]. Enzymes typically described in CD events in phytoplankton such as CL proteases are also implicated in managing the stress-response to UVR [18, 41], accordingly with some of the functions attributable to CLs in plants unrelated to CD [45]. The cleavage rate of CLs-reporter substrates was 10-fold lower than MCs-substrates. Unfortunately, there are no antibodies against CLs as such, due to the wide variety of enzymes encompassed within the CL group/terminology. There are aspartate-proteases and subtilases in *D. salina* genome, thus we suggest that CLs in *D. tertiolecta* are able to hydrolyse after aspartate in P1, and are possibly similar to other CLs already described in Viridiplantae, although their real nature is not unraveled yet. The high resilience and tolerance of *D. tertiolecta* to increased UVR are indicators that this species could be a benefited one and have the potential to become dominant during community shifts generated within the predicted future global change scenario. However, among the mechanistic questions underlying resilience, the role and activity of MCs have not been investigated so far in this species. MCs appear to be responsible for CD in phytoplankton [20]. Yet, we wonder whether MC-mediated proteolysis is directly responsible for morphological changes that occur during CD in non-metazoan organisms, or it is just one more of the versatile capacities of MCs. A key question is whether MCs are playing any role in the stress response in phytoplankton and other non-metazoan organisms, as well as in other physiological CD unrelated processes.

Immunoblot analysis demonstrated the unequivocal presence of Type-II MCs in *D. tertiolecta*. Type-II MCs are exclusively found in vascular plants and green algae [46] only encountered in the genomes of *Chlamydomonas reinhardtii, Volvox carteri, Chlorella sorokiniana* and *Tetraselmis* sp. up to date. They are absent in utmost other phytoplankton. Type-I MCs are also found in green algae and in nearly all eukaryotic phytoplankton [46]. In contrast, Type-III MCs and MC-like proteases are present in most eukaryotic phytoplankton, but not in plants and green algae [46]. The antibody that was raised against Arabidopsis MC9 (α-AtMC9) revealed one unique 60 KDa band of increasing intensity in parallel with progressive elevation of the UVR doses. It also cross-reacted with *A. thaliana* sub-type over-expressing MC9 (Atoe9) and MC1 (Atoe1) as positive controls **(Fig 2)**. The Dt-Type-II-MC molecular weight is higher than those MWs reported for *A. thaliana*, but this divergence is reasonable considering the eminent variability existing among the different MCs from *A. thaliana* itself [31], as well as between the diverse organisms grouped in the different kingdoms. The high affinity of this antibody for putative Dt-Type-II-MCs epitopes is demonstrated by the high dilution of the 1^ary^ and 2^ary^ antibodies used (the concentration of antibodies used in western blots show a reverse trend with the antibody affinity). The specificity of the antibody for Type II-MC epitopes was firstly proved with α-AtMC9 blocked with MC9 recombinant protein (RCMC9). It did not cross-react at all with any extracted proteins from *D. tertiolecta* or *A. thaliana*, because the recognition site had RCMC9 bound to it, and thus it could not bind to other MCs with their epitopes available.

Secondly, unspecific cross-reactions with any of the rubisco subunits are frequent because the antibodies that are raised against rubisco in the rabbit's diet might be present because of the heightened immune response during immunisation, and they react with the very abundant rubisco. In our case, the RCMC9-blocked antibody did not cross-react with rubisco protein, confirming the absence of unspecific cross-reactions with it. Moreover, when the α-AtMC9 was blocked with pure rubisco protein, it clearly recognised again the 60 KDa bands corresponding to *D. tertiolecta*, Atoe9 and Atoe1 **(Fig. 2)**, reinforcing α-AtMC9 specificity for Dt-Type-II-MCs. Immuno-isolation and co-precipitation of proteins from *D. tertiolecta* with α-AtMC9 evidenced a band of ≈ 60KDa corresponding to that obtained by western blotting, thus confirming that such band corresponded to a type-II MC (data not shown).

In the third place, membranes probed with the pre-immune sera as control were blank, corroborating that the proteins were not recognised by the antibody obtained from rabbits that had not been in contact with the antigen, therefore the possibility of another source for unspecific cross-reactions is eliminated. The use of a different specific 1^ary^ antibody against type II-MCs (α-AtII) also cross-reacted with proteins of *D. tertiolecta* showing a 26 KDa band of increasing intensity over time. The same controls as above were carried out, again evidencing the participation of Type-II-MCs in the UVR-induced stress response in *D. tertiolecta* **(Fig. 3)**.

Fourth, during massive CD of *D. tertiolecta* in darkness [27] Dt-Type-II-MCs accumulation pattern in western blots was opposed to the increment of dead cells **(Fig 4)**. Survival of the cells under UVR (i.e. same experiment as this work) occurred at the expense of growth [42] and preservation of cell viability was parallel to Type-II-MCs accumulation over the experimental time **(Fig. 2A)**.

Type-II-MCs in *D. tertiolecta* appear to be involved in the response to UVR. We assessed MCs activities by using specific fluorescent substrates and inhibition kinetics. The cleavage of all of the fluorogenic tri/tetra-peptide substrates with R or K residues at the P1 position **(Table 1)** used to detect possible MC activities in cellular lysates, was effectively inhibited by both reversible or irreversible serine protease, caspases, and cysteinyl proteases inhibitors **(Fig. 6B-F)** meaning that such proteins would not probably be MCs. One of the most compelling arguments in favour of the latter is that MCs activity is not blocked by caspase inhibitors [36]. A different outcome was observed with RVRRase escaping inhibition **(Fig. 6A)** and enzymatic activity sharply increasing over time **(Fig. 5C)**. One limitation of this method is the use of cell lysates with a protein pool and not a purified protein. This could mask putative activities, or may yield false positive results. In this case, one must assume that a fraction of the enzymatic pool can compete for the substrate and the inhibitor and then the enzymatic activity assessment might not be accurate. It is therefore compulsory to assay as many tri-or tetrapeptides with R or K in P1 as possible, to analyse the Michaelis-Menten kinetics in order to choose the right substrate concentration for the enzymatic assays, and running the enzymatic reactions with the proper irreversible inhibitors with Arg and Lys in P1. Otherwise, the assay of just one substrate would not be representative nor accurate, as it can be randomly degraded. We recommend the use of recombinant proteins product of gene cloning and expression, obtained from the cells under treatments to assay MC enzymatic activities, when possible. This is not always the case and alternative ways must be examined to keep on gaining insight into the underlying molecular mechanisms of proteases behaviour under stressful conditions. Unfortunately, we failed to clone any type-II and type I MC gene by using primers designed based on *D. salina*, and degenerate primers based on *D. salina, C. reinhardtii* and *A. thaliana* MCs conserved domains, and so we were not able to further analyse whether the accumulation of transcripts correlated with the enzymatic activities or what exactly was the activity of the expressed gene. However, due to the lack of inhibition of RVRRase by the various protease inhibitors described above, RVRRase is a potential candidate for being a MC.

We then synthetized the specific irreversible inhibitor Ac/Dec-RVRR-CMK which inhibited the activity by 95% at 29.5 μM **(Fig. 7)** pointing to RVRRase as a presumable MC. R/Kase enzymatic activities showed a steady state behaviour with non-significant differences between protease activity and/or time in *D. tertiolecta* dark-induced-CD **(Fig. 5E)**, implying that RVRRase activity was not involved in *D. tertiolecta* dark-induced CD, and supporting the data obtained by western blotting and flow cytometry, already suggesting that MCs are not involved in CD in this green alga.

All the potential MC activities were characterised from zymograms and by mass spectrometry analyses to unravel the identities of the enzymes associated with K/Rase activity. Kase activity was not detected in any zymogram, which is counterintuitive attending to Kase enzymatic activities measured *in vitro* **(Fig. 5C)**. Peptide mass spectrometry results suggest that the bands with Rase enzymatic activity obtained in *D. tertiolecta* and *A. thaliana* zymograms (B1 to B13) would not be MCs, which is equally in disagreement with the assayed enzymatic activities **(Fig. 5C)**.

Since α-AtMC9 and α-AtII antibodies showed elevated affinity and specificity for MCs epitopes in *D. tertiolecta*, one might expect that protein sequences in *D. salina* most likely present high similarity with *A. thaliana* due to low *D. salina* and *D. tertiolecta* SNP rate [44]. Based on the presence of type-II MCs specific conserved catalytic amino acid residues (i.e. HYSGHGT and CHSG) obtained after multiple sequence alignments of Type II MCs from *A. thaliana* and *D. salina* with Clustal Omega (EMBL-EBI), the protein Dusal.0158s00011.1 from *D. salina* showed the best E-value compared to type II MCs from *A. thaliana*, but also great similarity between putative type II-MCs and almost all type II-MCs from *A. thaliana* were found (Figure 1S-supplemental material). Such *D. salina* sequence was used to compare the protein fragments obtained from *D. tertiolecta* in the MALDITOF/TOF analyses and to check for significant homologous domains with putative MCs from *D. tertiolecta* by using Pfam, Interpro and Prosite ([47–49]). As expected, no typical metacaspase domains were present in any of the proteins obtained in the zymograms.

However, it is intriguing that some of the proteins identified in the zymograms by MALDITOF/TOF analyses are natural physiological substrates of MC in other Viridiplantae. For instance, the Tudor staphylococcal nuclease (TSN) protein found in B1 from *D. tertiolecta* **(Fig. 8B**, Table 2S-Supplemental material) was the first natural plant MC substrate identified in *Picea abies* [50] and it has also been reported to be a MC target of *A. thaliana* and *Populus* trees [51]. The glyceraldehyde-3-phosphate dehydrogenase found in bands B4 and B7 from *A. thaliana* **(Fig. 8B**, Supplemental Table 2S) is a physiological specific substrate of the yeast MC Yca1 [52]. More intriguing is that rubisco large subunit was found in QRR and GRR zymograms from *D. tertiolecta*; in RVRR, QRR and GRR zymograms from *A. thaliana* overexpressing MC9 and MC1 and *A. thaliana* wild type **(Fig. 8B**, Supplemental Table S2). Surprisingly, rubisco has not been clearly identified as a physiological substrate of MCs, although a small rubisco subunit has been found in *A. thaliana* MC9 degradome [32]. It is noticeable that rubisco was absent in the mass spectrometry analyses of RVRRase zymograms from *D. tertiolecta*, while it appeared in RVRRase zymograms bands from Atoe9 with extremely high fluorescent intensity, and also in Atoe1 with significantly less activity **(Fig. 8B**, Supplemental Table 2S). Rubisco is by far the most abundant protein on earth [53, 54], hence, it is reasonable to think that it can show up in mass spectrometry analysis as remaining fragments, witness of former degradation events, despite the fact that it has a very low turnover due to its high abundance and its constitutive feature within the cells. Still, this would not explain why the rubisco band was absent in RVRRase zymograms from *D. tertiolecta*. Our data are inconclusive regarding the identities of the proteins found by peptide-mass fingerprinting. Yet, it is also possible that highly abundant proteins such as rubisco co-migrate at the same molecular weight as RVRRases, masking the true responsible for the protease activities which are present.

## CONCLUDING REMARKS

This study demonstrates the presence of Type-II-MC(s) in the marine unicellular green alga *D. tertiolecta* (Viridiplantae) and their participation in the cellular stress response to ultraviolet exposure. Specifically, the following events occurred in parallel: (1) the accumulation pattern and specificity of the immunodetected Dt-Type-II MC shown in western blots over time, (2) the increased hydrolysis of Boc-RVRRase-AMC substrate along the experimental course, (3) the lack of RVRRase activity blockage by caspase inhibitors, (4) the full inhibition of RVRRase activity by the irreversible Ac/Dec-RVRR-CMK inhibitor and, (5) the lowest apparent Michaelis-Menten constant (K_M_^ap^) of all R/Kases observed corresponded to RVRRase, indicating elevated affinity by RVRR substrate; (6) the increased hydrolytic signal in RVRRase zymograms over time. Taken together our data strongly support the hypothesis that RVRRase enzymatic activity might be a type-II MC. In contrast, all of the rest of Rase and Kase activities were disqualified to be considered as MCs due to their unspecific behaviour during inhibition. Furthermore, RVRRase activity did not increase in *D. tertiolecta* dark-induced CD meanwhile immunodetected Dt-Type-II-MCs accumulation and increase of dead cells showed an inverse trend. This evidences that type-II MCs are not involved in CD, but rather in survival strategies under stressful irradiance conditions in this chlorophyte, in agreement with the heterogeneity of proteolytic functions described for MCs in plants [28, 30, 56]. Given the pleiotropic feature of MCs, we consider that measuring MCs activities alone is not sufficient to ascribe a death process where they intervene, as PCD. Although zymograms and peptide-mass fingerprinting revealed the identities of Rase activities suggesting an indirect evidence of possible natural physiological substrates of MCs, we cannot accurately conclude at present whether this is the case nor whether there are also type-I MCs involved in the response, and thus further investigation is required by using different proteomic-based approaches.

The present study highlights the influential ecological consequences that changes in stratospheric ozone levels may have on the responses of phytoplankton UVR exposure at the molecular level and how that is regulated. Phytoplankton is at the base of the food webs constituting a key component of biogeochemical cycles. Research on the stress response of phytoplankton at the molecular-ecophysiology level is essential in a rapidly changing environment consequence of global change, since in our planet, half of the organic carbon incorporation is due to phytoplankton [12]. Although caution is necessary and general responses cannot be ruled out from laboratory studies to field conditions, light structures biological communities and differential responses of phytoplankton species to fluctuating UVR can lead to shifts in species composition within natural populations, favouring species that are able to better cope with stressful irradiances. This constitutes a key component of species specificity within the actual global change scenario, ultimately regulating biodiversity, ecosystem stability and ecosystem services.

## MATERIALS AND METHODS

### Experimental set-up and culture conditions

Batch axenic cultures of the unicellular chlorophyte *D. tertiolecta* Butcher (CCAP 19 / 6B) originally isolated from a Norwegian fjord were grown in artificial seawater medium [55] enriched with f/2 nutrients [56], in sterile acrylic cylinders (Plexiglas XT^®^ 29080) transparent to UVR. Cultures were maintained at 16°C at 120 μmol quanta m^–2^ s^–1^ continuous irradiance. To avoid cell shading, cells were subjected to continuous stirring and aeration through 0.2 μm fiberglass filters (Millipore). When cultures reached mid-log phase, they were verified for bacterial contamination by using DAPI (Molecular Probes, Oregon, USA) according to [15], and the cells were then exposed to PAR+UVA+UVB (PAB) for six days (144 h). Sampling started after switching the UVR on. Cells were harvested every 24 h. The stressful UVR effect was assayed in four independent replicate cultures (N=4) and samples from each culture cylinder were analysed for each variable aim of study.

The irradiance conditions were achieved by covering the experimental cylinders with an Ultraphan-295 UBT 500 mm cut-off filter (Digefra, Munchen, Germany) which transmitted PAR, UVA, and UVB (PAB). This filter has no transmission below 295 nm (UVC). Since UVC does not reach the Earth's surface, it is not realistic to include the UVC band in any experimental set-up studying environmental responses. PAR irradiance at 120 μmol photons m^-2^ s^-1^ was obtained by using Optimarc 250W lamps (DuroTest, USA) and measured using an Ocean Optics SMS 500 spectroradiometer (Sphaereoptics, New Hampshire, USA) calibrated after NPL standards with a cosine-corrected sensor. Experimental UVR fluence rates were provided by Qpanel-340 lamps simulating the natural solar radiation conditions reaching the upper photic zone and measured with an Ocean Optics SMS 500 spectroradiometer mentioned above. Spectra were measured in the range 200–800 nm. All light measurements were carried out inside the experimental cylinders once they were wrapped with the UVC cut-off filter. Experimental irradiances (unweighted) were 9.5 Wm^-2^ UVA and 0.5 Wm^-2^ UVB (UVR:PAR ratio =0.38) according to [42]. The experimental PAR:UVR ratio was specifically chosen to be able to analyse the contribution of total UVR on the physiological response, without being masked by both the effects of high PAR and UVA and UVB wavelengths separation. For comparison with other studies, weighted irradiances corresponding to the measured light spectra were the same as those calculated by [18] by using the appropriate biological weighting functions (BWFs).

### Cell abundance and *in vivo* chlorophyll a fluorescence

Cellular density was determined in 1 mL fresh samples by flow cytometry (Accuri, BD, USA) under blue excitation with chl a as the trigger according to [57]. The maximal quantum yield of Photosystem II (PSII) fluorescence (F_v_/F_m_) was measured with a Water-PAM fluorometer (Waltz, Effeltrich, Germany) as described in [41]. High F_v_/F_m_ values indicate that cells are in good physiological condition, whereas a decrease of F_v_/F_m_ indicates stress and/or photoinhibition.

### MCs immunodetection.

For MCs detection and accumulation, SDS- PAGE was performed according to [27] on an equal protein concentration loading basis. For MCs immunodetection and band analyses, western blots were probed with polyclonal antibodies against type I and type II MCs from the model plant *A. thaliana* [58]. The antibody against type I MC (α-AtI) was checked for cross reactivity at 1:7000 dilution and subsequently incubated with an anti-rabbit secondary antibody at 1:15000 dilution. The antibody crossing with all type II MCs (α-AtII) and the antibody specific against MC9 (α-AtMC9) were used at 1:10000 dilution and subsequently incubated with an α-rabbit 2^ary^ antibody at 1:20000 dilution.

The following controls were carried out to check for specificity and affinity of the antibodies: (1) each 1^ary^ antibody was substituted with its correspondent pre-immune sera at the same dilution than the used 1^ary^ and 2^ary^ antibodies described just above; (2) as positive controls, proteins extracted from *A. thaliana* wild type leaves and from two sub-types of *A. thaliana* over-expressing MC9 (Atoe9, [58]) and over-expressing MC1 ( Atoe1, [59]) were electrophoresed and western blotted together with *D. tertiolecta* samples incubated in PAB during 48h; (3) the MC9 recombinant protein [31] was used as a positive control and as the α-AtMC9-blocking peptide to ensure absolute specificity of the 1^ary^ α-AtMC9. The blockage to binding site ratio was 1:4 (antibody/pure recombinant protein) in moles; (4) primary antibodies were also blocked with commercial pure rubisco (Sigma-Aldrich) to ensure no recognition of this protein when using the 2^ary^ α-rabbit antibodies; (5) a secondary antibody non-specific cross-reactivity control was carried out by incubating the membranes with only the 2^ary^ antibody in the absence of the 1^ary^ antibody; (6) dark-treated *D. tertiolecta* samples were used as a positive control for CD as described in [57]. The intensity of cross-reactions was quantified by chemiluminescence (ECL-Advanced; GE Healthcare) in a Gel Logic Image Analyser (Eastman-Kodak, Rochester, NY).

### CL and MC enzymatic activities

Cells (50 mL) were harvested by centrifugation, resuspended in 2 mL lysis buffer containing 50 mM HEPES (pH 7.3), 100 mM NaCl, 10% sucrose, 0.1% 3-[(3-cholamidopropyl)-dimethylammonio]-1-propanesulphonate (CHAPS), and 10 mM dithiothreitol, and sonicated (UP50H, Hielscher GmbH, Germany) on ice 3 x 10s allowing 10 min recovery after each cycle. Cell lysates were mixed with different concentrations of Boc-7-amino-4-fluoromethyl coumarin (AMC) reporters of the substrates QRR, GRR, LKR, RVRR, HEK, and VLK (catalogue # 3122-v, 3142-v3141-v, 3155-v, 3215-v, 3104-v, respectively, Peptanova GmbH, Germany) were used to detect potential MCs. Ac-7-amino-4-trifluoromethyl coumarin (AFC) reporters of the substrates WEHD, DEVD, IETD, and LETD (catalogue # 3186v, 3171v, 3195v, and 3198v, respectively, Peptanova GmbH, Germany) were used to detect CL activities. The specific aminoacidic sequences of the substrates can be found in **Table 1**. The emitted fluorescence was measured in the kinetic mode for 4 h at 16°C (excitation 360 nm, emission 460 nm) in a microplate fluorescence reader (FL-600, BIO-TEK, USA). The total protein content was measured by using the bicinchoninic acid assay (BCA) [60]. The enzymatic activity was expressed as relative fluorescence units per time and per equal protein content (RFU s^-1^ prot^-1^).

To measure the enzymatic activities accurately over the experimental time, the optimal substrate concentration had to be determined first. For this, enzymatic assays were carried out by incubating the cell extracts of 48 h under PAB with 25, 50, 100, 200 and 400 μM of each of the reporter substrates. A Michaelis-Menten kinetic was modelled to calculate the apparent Michaelis-Menten constant (K_M_^ap^). The term “apparent” is adopted when K_M_ is observed under conditions that would hinder the determination of its true value (e.g. the presence of a competitive substrate/ reversible inhibitor of any kind). Due to our enzymatic activity being measured in cell lysates and not in purified proteins, we assume K_M_^ap^ as the correct parameter.

### Inhibition of the enzymatic activity

To get a better insight on the enzyme kinetics for the MCs reporter substrates, samples exposed to PAB during 48 h were incubated for 1 h at 16°C (previously to the activity assay) with the following reversible and irreversible protease inhibitors, at the indicated final concentrations: (1) benzamidine at 100 μM; (2) phenylmethylsulfonyl fluoride (PMSF) at 100 μM; (3) benzyloxycarbonyl-Val-Ala-Asp (OMe) fluoromethylketone (Z-VAD-FMK) at 100 and 50 μM; (4) 4A-phenylmethylsulfonyl fluoride (4A-PMSF) at 100 and 50 μM; (5) benzyloxycarbonyl Asp (OMe) fluoromethylketone (Boc-D-FMK) at 100 and 50 μM; (6) trans-Epoxysuccinyl-L-leucylamido(4-guanidino) butane (E64) at 200 and 75 μM; (7) Leupeptin at 100 and 50 μM. Detailed inhibition target groups can be consulted in Table S1-Supplemental material.

### Synthesis of Ac/Dec-RVRR-CMK irreversible inhibitors and RVRRase inhibition kinetics

One of the most striking results obtained in the enzyme kinetics assays was the behaviour of RVRRase activity. Due to the lack of commercial RVRR inhibitors and our need of studying its inhibition, we synthesized two irreversible RVRRase inhibitors. For these syntheses, a convergent strategy was employed according to which two peptidic fragments (A/A' and B) were synthetized separately and then coupled. The peptidic chain contained in fragments A and A' was prepared by using solid phase methodology on a 2-chlorotrityl chloride (CTC) resin [61] applying the Fmoc strategy [62] as follows: the CTC resin was properly derivatized with Fmoc-Arg (Boc)_2_-OH using N,N-diisopropylethylamine (DIPEA) in N,N-dimethylformamide (DMF). Then, the Fmoc (9-fluorenylmethoxycarbonyl) protecting group was removed by treatment with 20% solution of piperidine in DMF and the following Fmoc protected aminoacid, Fmoc-Val-OH, was loaded onto the resulting resin by the action of N,N'-diisopropylcarbodiimide (DIC) in the presence of 1-hydroxybenzotriazole (HOBt) in DMF. The same sequence of Fmoc deprotection and peptidic coupling was repeated twice to introduce sequentially a new unit of Fmoc-Arg (Boc)_2_-OH and CH_3_COCl for A or CH_3_(CH_2_)_8_COCl for A'. At the end of the solid phase peptide synthesis, the resin-bound peptide was cleaved from the resin by treatment with a AcOH:TFE:CH_2_Cl_2_ (7:2:1) mixture to obtain the fragments A and A', respectively. Fragment B was prepared as described by [63] as follows: Commercially available Boc-Arg (Mtr)-OH was treated with isopropyl chloroformate in the presence of 4-methylmorpholine followed by reaction with CH_2_N_2_ in Et_2_O to obtain the corresponding diazo derivative. Final treatment with methanolic HCl afforded the fragment B. Fragments A or A' and B were coupled by using DIC, HOBt and DIPEA to obtain the corresponding peptides, which were finally subjected to a treatment with a solution of trifluoroacetic acid (TFA) in MeOH to remove the Mtr (2,3,6-trimethyl-4-methoxybenzenesulfonyl ) and Boc (tert-butyloxycarbonyl) protecting groups to yield the peptides Ac-Arg-Val-Arg-Arg-CMK and Dec-Arg-Val-Arg-Arg-CMK (Ac/Dec-RVRR-CMK).

The inhibition kinetics was assayed according to the Easson & Stedman model [64] for the calculation of the apparent inhibition constant (K_i_^ap^) when using irreversible inhibitors. Samples exposed to 48h PAB were pre-incubated with 10, 20, 40, 80 and 160 μM of the inhibitor (final concentrations) for 1h. The RVRRase activity was then measured by adding 50 μM of Boc-RVRR-AMC substrate according to the optimal substrate concentration obtained previously in the Michaelis-Menten kinetics assay. The substitution of cell extracts by buffer and no addition of RVRR substrate to the cell extracts in the reaction mix were used as negative controls.

### Zymograms and peptide-mass-fingerprinting analyses

SDS-PAGE was carried out as described previously and gels were renaturalised according to [65 and references within] to obtain active enzymes. For this purpose, gels were incubated twice in 0.05 M Tris–HCl buffer with 20 % Triton X-100 at pH 7.4 for 10 min. Subsequently, they were again incubated twice in the same buffer without Triton X-100 for 10 min. The reconstitution of the enzymatic activity is possible because the denaturation agents transforms proteins into a random coil conformation and the removal of these agents is accompanied by the recovery of the native structure, therefore activity. The enzymatic activities were detected and quantified. Gels were incubated in the reaction buffer containing each of the AMC-reporters of the substrates QRR, GRR, LKR, RVRR, HEK, and VLK described in the enzymatic activities assay section (one gel, one substrate) overnight at 4°C. Gels were analysed in the image analyser by using a 535 nm detection filter. The recovery of the enzymatic activity was always tested by comparing the in-gel activities to native protein extracts incubated with the appropriate fluorescent substrates and it was never lower than 95%.

The protein bands were excised from the gel and electroeluted by using an electrophoretic-protein-concentration device ISCO 1750 (ISCO Inc., Lincoln, NE). Electroelution was performed through 10 KDa pore dialyses membranes in 25 mM Tris –Gly buffer with 0.1% SDS at pH 8.3. The electroeluted proteins were digested with trypsin. The generated fragments were analysed by nanoscale liquid chromatography (nano LC) coupled to peptide mass fingerprinting by using a MALDI (matrix-assisted laser desorption ionization) source and tandem time-of-flight (TOF/TOF) mass analysers (4700 Proteomics Analyzer, Applied Biosystems). For protein identification, peptide lists from the MS analysis were submitted to the Mascot search engine version 2.1 (Matrix Science, London, UK) integrated in GPS ExplorerTM v3.5 (AB Sciex). A combined (MS+MS/MS) type analysis was run by aligning similar fragments with those sequences uploaded in NCBInr Uniprot databases restricted to Viridiplantae as search criteria.

### Statistical analyses

Data were checked for normality by Shapiro-Wilks' test and homoscedasticity by Cochran's and Levene's tests. Variables met all criteria to perform parametric tests. Any significant influence of the cylinders for the cultures was discarded by a nested ANOVA (p>0.05). Statistical significance of treatment effects was analysed by using 2 Way-ANOVA followed by post-hoc Sidak, Tukey or Newman-Keuls (considering p<0.05 and/or p<0.01 as significant). All analyses were performed by using the GLM (general linear model) procedure with main effects and interaction terms. When appropriate the three pseudoreplicates samples from each cylinder were considered replicates, since the nested ANOVA was not significant, and so the mean of the values was used. Statistical analyses were carried out by using the software Statistica v12 (Statsoft, Inc.).

## SUPPLEMENTAL MATERIAL

Click here for supplemental data file.

All supplemental data for this article are available online at http://microbialcell.com/researcharticles/2019a-mata-microbial-cell.

## Abbreviations:

CD: – cell death,
CFC: – chlorofluorocarbons,
CL: – caspase-like,
MC: – metacaspase,
PAB: – PAR + UVA + UVB,
PAR: – photosynthetic active radiation,
PCD: – programmed CD,
UV: – ultraviolet
UVR: – UV radiation.
