# Supplementary material for: Type II-Metacaspases are involved in cell stress but not in cell death in the unicellular green alga Dunaliella tertiolecta
Source: Microb Cell. 2019 Oct 7;6(11):494–508. doi: 10.15698/mic2019.11.696 (PMC6859423; doi:10.15698/mic2019.11.696)
Supplement: Supplementary file 1 [file mic-06-494-s01.pdf]

Supplemental material

Type II-Metacaspases are involved in cell stress but not in cell death in the unicellular green algae *Dunaliella tertiolecta*

M. Teresa Mata[1&], Armando Palma[1], Candela García-Gómez[1*#], María López-Parages[1], Víctor Vázquez[1], Iván Cheng-Sánchez[2], Francisco Sarabia[2], Félix López-Figueroa[1], Carlos Jiménez[1], María Segovia[1*]

Table S1. Inhibition of Rase and Kase enzymatic activities with reversible and irreversible protease inhibitors presenting different target inhibition sites, at different final concentrations.

**Inhibitor**	**Compound**	**Target protoein inhibition**	**Type of inhibition**
**PMSF**	Phenylmethylsulfonyl fluoride	Serine-proteases	Reversible
**Z-VAD-FMK**	Benzyloxycarbonyl-Val-Ala-Asp (OMe) fluoromethylketone	Caspases	Irreversible
**4A-PMSF**	4-(2-Aminoethyl)benzenesulfonyl fluoride hydrochloride	Serin-proteases	Irreversible
**BOC-D-FMK**	Boc-Asp(OMe) fluorometylketone	Caspases	Irreversible
**E64**	*Trans*-Epoxysuccinyl-L-leucylamido(4-guanidino) butane	Cisteinyl-proteases	Irreversible
**Leupeptin**	Leupeptin	Serine & cisteinyl-proteases	Reversible
**Benzamidine**	Benzamidine	Poly-ADPribose polymerase (PARP)	Reversible

Table S2. In-gel-Rase activity bands (zymograms) analyses by MALDI (matrix-assisted laser desorption ionization) source and tandem time-of-flight (TOF/TOF) mass analyses. For protein identification, peptide lists from the MS analysis were submitted to the Mascot search engine version 2.1 (Matrix Science, London, UK) integrated in GPS ExplorerTM v3.5 (AB Sciex). A combined (MS+MS/MS) type analysis was run by aligning similar fragments with those sequeces uploaded in NCBInrUniprot databases restricted to Viridiplantae as search criteria. For each Rase substrate the 10 most probable matching proteins found are shown. The protein score C.I. % is the confidence interval for the protein scores that the Mascot search engine assigns to each identification. Values above 99% are considered as significant high identity.

Zymogram	Band/Species	Protein Score C.I.%	Protein name	M.W. (KDa)	Accession number
**RVRR**	1/*D.t*	99.812	**Phytochrome B, partial [*Tarenaya hassleriana*]**	117.1	AAW56607.1
	1/*D.t*	99.746	**Homeodomain-like superfamily protein [*Arabidopsis thaliana*]**	447.1	NP_974216.1
	1/*D.t*	99.598	**110 kDa 4SNc-Tudor domain protein [*Pisum sativum*]**	108.6	BAC06184.1
	1/*D.t*	99.198	**Asparaginyl-tRNA synthetase, partial [*Hyacinthus orientalis*]**	10.0	AAT08691.1
	1/*D.t*	99.058	**Hypothetical protein PHYPA_024096 [*Physcomitrella patens*]**	78.6	PNR34279.1
	1/*D.t*	98.918	**THO complex subunit 4A-like isoform X1 [*Physcomitrella patens*]**	28.9	XP_024365628.1
	1/*D.t*	98.841	**Unknown protein [*Arabidopsis thaliana*]**	13.9	AAD23025.2
	1/*D.t*	98.541	**CDT1-like protein a, chloroplastic [*Oryza sativa Japonica* Group]**	64.5	XP_015634451.1
	1/*D.t*	97.345	**Hypothetical protein VITISV_010874 [*Vitis vinifera*]**	37.3	CAN82058.1
	1/*D.t*	97.22	**Retrotransposon protein, putative, Ty3-gypsy subclass [*Oryza sativa Japonica* Group]**	17.3	ABA96803.1
**RVRR**	2/*D.t*	99.794	**PREDICTED: uncharacterized protein LOC100248586 [*Vitis vinifera*]**	54.4	XP_002267983.1
	2/*D.t*	98.893	**Protein PHOSPHATE STARVATION RESPONSE 3-like [*Physcomitrella patens*]**	62.1	XP_024363632.1
	2/*D.t*	98.507	**Micronuclear linker histone polyprotein, putative [*Solanum bulbocastanum*]**	17.6	AAP45160.2
	2/*D.t*	98.076	**(-)-limonene synthase [*Picea abies*]**	73.4	AAS47694.1
	2/*D.t*	96.733	**PREDICTED: protein yippee-like At4g27745 [*Oryza brachyantha*]**	12.7	XP_006662984.1
	2/*D.t*	94.323	**Kinesin-like protein KIN-12F [*Physcomitrella patens*]**	309.8	XP_024374937.1
	2/*D.t*	93.33	**Disease resistance gene analog, partial [*Zizania latifolia*]**	18.6	ABB69771.1
	2/*D.t*	89.429	**Uncharacterized protein LOC4336466 isoform X1 [*Oryza sativa* Japonica Group]**	15.1	XP_015633835.1
	2/*D.t*	89.429	**Hypothetical protein VITISV_024824 *[Vitis vinifera*]**	58.3	CAN61722.1
	2/*D.t*	88.139	**Pentatricopeptide repeat-containing protein At4g18520, chloroplastic-like [*Physcomitrella patens*]**	57,5	XP_024379060.1

**RVRR**	3/$A.t_{oe1}$	100	**Ribulose-1,5-bisphosphate carboxylase/oxygenase large subunit (chloroplast) [*Arabidopsis thaliana*]**	53.4/53.0	NP_051067.1
	3/$A.t_{oe1}$	100	**Large subunit of riblose-1,5-bisphosphate carboxylase/oxygenase (chloroplast) [*Arabis hirsuta*]**	53.4/53.0	YP_001123207.1
	3/$A.t_{oe1}$	100	**Ribulose-1,5-bisphosphate carboxylase/oxygenase large subunit (chloroplast) [*Olimarabidopsis pumila*]**	53.4/53.0	YP_001123123.1
	3/$A.t_{oe1}$	100	**Ribulosebisphosphate carboxylase, partial [*Arabidopsis thaliana*]**	48.0	BAA19595.1
	3/$A.t_{oe1}$	100	**Ribulose-1,5-bisphosphate carboxylase/oxygenase large subunit, partial (chloroplast) [*Crucihimalaya himalaica*]**	48.0/47.6	BAA19596.1
	3/$A.t_{oe1}$	100	**Ribulose-1,5-bisphosphate carboxylase/oxygenase large subunit, partial (chloroplast) [*Polanisia dodecandra*]**	49.9/49.6	AAO38787.1
	3/$A.t_{oe1}$	100	**Ribulose-1,5-bisphosphate carboxylase/oxygenase large subunit, partial (chloroplast) [*Heliophila pubescens*]**	52.0/51.5	CAJ86804.1
	3/$A.t_{oe1}$	100	**Ribulose-1,5-bisphosphate carboxylase/oxygenase large subunit, partial (chloroplast) [*Tarenaya hassleriana*]**	52.1/51.6	AAA84103.2
	3/$A.t_{oe1}$	100	**Ribulose-1,5-bisphosphate carboxylase/oxygenase large subunit, partial (chloroplast) [*Aethionema grandiflorum*]**	49.8/49.4	AAO38786.1
	3/$A.t_{oe1}$	100	**Ribulose-1,5-bisphosphate carboxylase/oxygenase large subunit, partial (chloroplast) [*Rorippa indica*]**	48.0/47.6	BAA19601.1
**RVRR**	4/$A.t_{oe1}$	100	**Glyceraldehyde-3-phosphate dehydrogenase C2 [*Arabidopsis thaliana*]**	36.9	NP_172801.1
	4/$A.t_{oe1}$	100	**RecName: Full=Glyceraldehyde-3-phosphate dehydrogenase, cytosolic**	36.9	P04796.2
	4/$A.t_{oe1}$	100	**Glyceraldehyde-3-phosphate dehydrogenase C subunit (GapC) [*Arabidopsis thaliana*]**	37.0	AAM65189.1
	4/$A.t_{oe1}$	100	**Glyceraldehyde-3-phosphate dehydrogenase C2 [*Arabidopsis thaliana*]**	33.9	NP_001077530.1
	4/$A.t_{oe1}$	100	**Glyceraldehyde-3-phosphate dehydrogenase, partial [*Pachycladon cheesemanii*]**	14.5	ABM98111.1
	4/$A.t_{oe1}$	100	**Cytosolic glyceraldehyde-3-phosphate dehydrogenase, partial [*Leavenworthia crassa*]**	13.5	AAC32382.1
	4/$A.t_{oe1}$	100	**Glyceraldehyde-3-phosphate dehydrogenase, partial [*Pachycladon radicatum*]**	14.5	ABM98114.1
	4/$A.t_{oe1}$	100	**Glyceraldehyde-3-phosphate dehydrogenase, partial [*Brassica napus*]**	17.6	AAZ05899.1
	4/$A.t_{oe1}$	100	**Glyceraldehyde-3-phosphate dehydrogenase, partial [*Brassica rapa subsp. Pekinensis*]**	18.5	AAN05699.1
	4/$A.t_{oe1}$	100	**Glyceraldehyde-3-phosphate dehydrogenase, partial [*Leavenworthia crassa*]**	8.5	AAC34080.1
**RVRR**	5/$A.t_{oe1}$	100	**Ribulose-1,5-bisphosphate carboxylase/oxygenase large subunit (chloroplast) [*Arabidopsis thaliana*]**	53.0	NP_051067.1

	5/*A.t*$_{oe1}$	100	**Ribulose-1,5-bisphosphate carboxylase/oxygenase large subunit, partial (chloroplast) [*Arabidopsis lyrata subsp. Lyrata*]**	47.5	AAO19421.1
	5/*A.t*$_{oe1}$	100	**Ribulose-1,5-bisphosphate carboxylase/oxygenase large subunit, partial (chloroplast) [*Lepidium africanum*]**	48.4	CAJ86805.1
	5/*A.t*$_{oe1}$	100	**Ribulose-1,5-bisphosphate carboxylase/oxygenase large subunit, partial (chloroplast) [*Erysimum capitatum*]**	49.5	AAO38783.1
	5/*A.t*$_{oe1}$	100	**Ribulose-1,5-bisphosphate carboxylase/oxygenase large subunit, partial (chloroplast) [*Aethionema saxatile*]**	51.6	AAS88130.1
	5/*A.t*$_{oe1}$	100	**Large subunit of riblose-1,5-bisphosphate carboxylase/oxygenase (chloroplast) [*Arabis hirsuta*]**	53.0	YP_001123207.1
	5/*A.t*$_{oe1}$	100	**Large subunit of riblose-1,5-bisphosphate carboxylase/oxygenase (chloroplast) [*Draba nemorosa*]**	53.0	YP_001123558.1
	5/*A.t*$_{oe1}$	100	**Large subunit of riblose-1,5-bisphosphate carboxylase/oxygenase (chloroplast) [*Aethionema grandiflorum*]**	53.7	YP_001123038.1
	5/*A.t*$_{oe1}$	100	**Ribulose-1,5-bisphosphate carboxylase/oxygenase large subunit, partial (chloroplast) [*Aethionema grandiflorum*]**	49.4	AAO38786.1
	5/*A.t*$_{oe1}$	100	**Ribulose-1,5-bisphosphate carboxylase/oxygenase large subunit, partial (chloroplast) [*Chamira circaeoides*]**	50.0	CAJ86803.1
**GRR**	6/*A.t*$_{oe1}$	100	**Ribulose-1,5-bisphosphate carboxylase/oxygenase large subunit (chloroplast) [*Arabidopsis thaliana*]**	53.0	NP_051067.1
	6/*A.t*$_{oe1}$	100	**Large subunit of riblose-1,5-bisphosphate carboxylase/oxygenase (chloroplast) [*Draba nemorosa*]**	53.0	YP_001123558.1
	6/*A.t*$_{oe1}$	100	**Ribulosebisphosphate carboxylase, partial [*Arabidopsis thaliana*]**	47.6	BAA19595.1
	6/*A.t*$_{oe1}$	100	**Ribulose-1,5-bisphosphate carboxylase/oxygenase large subunit (chloroplast) [*Olimarabidopsis pumila*]**	52.9	YP_001123123.1
	6/*A.t*$_{oe1}$	100	**Ribulose-1,5-bisphosphate carboxylase/oxygenase large subunit, partial (chloroplast) [*Limnanthes douglasii*]**	52.1	AAA84354.2
	6/*A.t*$_{oe1}$	100	**Ribulose-1,5-bisphosphate carboxylase/oxygenase large subunit, partial (chloroplast) [*Scutellaria alpina*]**	52.4	CAA85705.1
	6/*A.t*$_{oe1}$	100	**Ribulose-1,5-bisphosphate carboxylase/oxygenase large subunit, partial (chloroplast) [*Sebaea sp*. Forest 560]**	51.6	CAJ86855.1
	6/*A.t*$_{oe1}$	100	**Ribulose-1,5-bisphosphate carboxylase/oxygenase large subunit, partial (chloroplast) [*Aethionema saxatile*]**	51.6	AAS88130.1

	6/*A.t*$_{oe1}$	100	**Ribulose-1,5-bisphosphate carboxylase/oxygenase large subunit (chloroplast) [*Crucihimalaya wallichii*]**	53.0	YP_001123470.1
	6/*A.t*$_{oe1}$	100	**Large subunit of riblose-1,5-bisphosphate carboxylase/oxygenase (chloroplast) [*Aethionema cordifolium*]**	53.3	YP_001122954.1
**GRR**	7/*A.t*$_{oe1}$	100	**Glyceraldehyde-3-phosphate dehydrogenase C subunit 1 [*Arabidopsis thaliana*]**	36.9	NP_187062.1
	7/*A.t*$_{oe1}$	100	**Glyceraldehyde-3-phosphate dehydrogenase C2 [*Arabidopsis thaliana*]**	33.9	NP_001077530.1
	7/*A.t*$_{oe1}$	100	**Recname: Full=Glyceraldehyde-3-phosphate dehydrogenase; Short=GAPDH**	39.1	P34783.1
	7/*A.t*$_{oe1}$	100	**Putative glyceraldehyde-3-phosphate dehydrogenase [*Arabidopsis thaliana*]**	17.3	BAD94800.1
	7/*A.t*$_{oe1}$	100	**Putative cytosolic fructose-bisphosphate aldolase, partial [*Populus x canadensis*]**	8.8	ABD35813.1
	7/*A.t*$_{oe1}$	100	**Glyceraldehyde-3-phosphate dehydrogenase C subunit, partial [*Arabidopsis thaliana*]**	8.7	BAD94315.1
	7/*A.t*$_{oe1}$	100	**Recname: Full=Glyceraldehyde-3-phosphate dehydrogenase, cytosolic**	36.9	P04796.2
	7/*A.t*$_{oe1}$	100	**Aldolase superfamily protein [*Arabidopsis thaliana*]**	38.4	NP_181187.1
	7/*A.t*$_{oe1}$	100	**Aldolase superfamily protein [*Arabidopsis thaliana*]**	28.7	NP_001118453.1
	7/*A.t*$_{oe1}$	100	**Glyceraldehyde-3-phosphate dehydrogenase C subunit (gapc) [*Arabidopsis thaliana*]**	37.0	AAM65189.1
**GRR**	8/*A.t*$_{oe1}$	100	**Ribulose-1,5-bisphosphate carboxylase/oxygenase large subunit (chloroplast) [*Arabidopsis thaliana*]**	53.0	NP_051067.1
	8/*A.t*$_{oe1}$	100	**Ribulose-1,5-bisphosphate carboxylase/oxygenase large subunit, partial (chloroplast) [*Aethionema saxatile*]**	51.6	AAS88130.1
	8/*A.t*$_{oe1}$	100	**Ribulose-1,5-bisphosphate carboxylase/oxygenase large subunit (chloroplast) [*Capsella bursa-pastoris*]**	53.0	YP_001123381.1
	8/*A.t*$_{oe1}$	100	**Large subunit of riblose-1,5-bisphosphate carboxylase/oxygenase (chloroplast) [*Draba nemorosa*]**	53.0	YP_001123558.1
	8/*A.t*$_{oe1}$	100	**Ribulose-1,5-bisphosphate carboxylase/oxygenase large subunit (chloroplast) [*Crucihimalaya wallichii*]**	53.0	YP_001123470.1
	8/*A.t*$_{oe1}$	100	**Large subunit of riblose-1,5-bisphosphate carboxylase/oxygenase (chloroplast) [*Aethionema cordifolium*]**	53.3	YP_001122954.1
	8/*A.t*$_{oe1}$	100	**Large subunit of riblose-1,5-bisphosphate carboxylase/oxygenase (chloroplast) [*Aethionema grandiflorum*]**	53.7	YP_001123038.1
	8/*A.t*$_{oe1}$	100	**Ribulose-1,5-bisphosphate carboxylase/oxygenase large subunit, partial (chloroplast) [*Chamira circaeoides*]**	50.0	CAJ86803.1
	8/*A.t*$_{oe1}$	100	**Ribulose-1,5-bisphosphate carboxylase/oxygenase large subunit, partial (chloroplast) [*Heliophila pubescens*]**	51.5	CAJ86804.1

	8/*A.t*$_{oe1}$	100	**Ribulose-1,5-bisphosphate carboxylase/oxygenase large subunit (chloroplast) [*Olimarabidopsis pumila*]**	53.0	YP_001123123.1
**QRR**	9/*D.t*	100	**Large subunit of riblose-1,5-bisphosphate carboxylase/oxygenase (chloroplast) [*Aethionema arabicum*]**	53,4	YP_009356788.1
	9/*D.t*	100	**Large subunit of riblose-1,5-bisphosphate carboxylase/oxygenase [*Arabis hirsuta*]**	52.9	YP_001123207.1
	9/*D.t*	100	**Ribulose-1,5-bisphosphate carboxylase/oxygenase large subunit (chloroplast) [*Capsella bursa-pastoris*]**	53,0	YP_001123381.1
	9/*D.t*	100	**Large subunit of riblose-1,5-bisphosphate carboxylase/oxygenase (chloroplast) [*Aethionema grandiflorum*]**	53.7	YP_001123038.1
	9/*D.t*	100	**Large subunit of riblose-1,5-bisphosphate carboxylase/oxygenase (chloroplast) [*Draba nemorosa*]**	52.9	YP_001123558.1
	9/*D.t*	100	**Ribulose-1,5-bisphosphate carboxylase/oxygenase large subunit, partial (chloroplast) [*Arabidopsis thaliana*]**	51.9	AMR44347.1
	9/*D.t*	100	**Ribulose-1,5-bisphosphate carboxylase/oxygenase large subunit, partial (chloroplast) [*Crucihimalaya himalaica*]**	47.6	BAA19596.1
	9/*D.t*	100	**Ribulose-1,5-bisphosphate carboxylase/oxygenase large subunit, partial (chloroplast) [*Rorippa indica*]**	47.6	BAA19601.1
	9/*D.t*	100	**Ribulose-1,5-bisphosphate carboxylase/oxygenase large subunit, partial (chloroplast) [*Lepidium africanum*]**	69.6	CAJ86805.1
	9/*D.t*	100	**Ribulosebisphosphate carboxylase, partial (chloroplast) [*Armoracia rusticana*]**	49.5	AAB71403.1
**QRR**	10/*D.t*	99.363	**PREDICTED: dexh-box ATP-dependent RNA helicase dexh10 isoform X1 [*Vitis vinifera*]**	112.6	XP_002266524.2
	10/*D.t*	99.252	**Hypothetical protein VITISV_035836 [*Vitis vinifera*]**	101.8	CAN80939.1
	10/*D.t*	97.986	**THO complex subunit 2-like isoform X1 [*Physcomitrella patens*]**	191.7	XP_024388620.1
	10/*D.t*	97.464	**Unknown [*Picea sitchensis*]**	19.4	ABK26501.1
	10/*D.t*	97.345	**Unnamed protein product [*Arabidopsis thaliana*]**	26.0	BAB11390.1
	10/*D.t*	96.808	**Putative transcription factor, partial [*Platanus x hispanica*]**	21.8	CAL25350.1
	10/*D.t*	96.162	**DEAD-box ATP-dependent RNA helicase 39 isoform X1 [*Oryza sativa* Japonica Group]**	68.2	XP_015613578.1
	10/*D.t*	95.056	**Hypothetical protein VITISV_040528 [*Vitis vinifera*]**	48.5	CAN78754.1
	10/*D.t*	94.94	**Hypothetical protein PHYPA_012615, partial [*Physcomitrella patens*]**	107.9	PNR48141.1
	10/*D.t*	94.94	**Hypothetical protein VITISV_031913 [*Vitis vinifera*]**	190.1	CAN71787.1
**QRR**	11/*A.t*$_{oe1}$	100	**Ribulose-1,5-bisphosphate carboxylase/oxygenase large subunit (chloroplast) [*Arabidopsis thaliana*]**	53.0	NP_051067.1

	11/*A.t*$_{oe1}$	100	**Large subunit of riblose-1,5-bisphosphate carboxylase/oxygenase (chloroplast) [*Draba nemorosa*]**	53.0	YP_001123558.1
	11/*A.t*$_{oe1}$	100	**Ribulose-1,5-bisphosphate carboxylase/oxygenase large subunit, partial (chloroplast) [*Aethionema saxatile*]**	51.6	AAS88130.1
	11/*A.t*$_{oe1}$	100	**Large subunit of riblose-1,5-bisphosphate carboxylase/oxygenase (chloroplast) [*Arabis hirsuta*]**	53.0	YP_001123207.1
	11/*A.t*$_{oe1}$	100	**Ribulose-1,5-bisphosphate carboxylase/oxygenase large subunit (chloroplast) [*Capsella bursa-pastoris*]**	53.0	YP_001123381.1
	11/*A.t*$_{oe1}$	100	**Large subunit of riblose-1,5-bisphosphate carboxylase/oxygenase (chloroplast) [*Aethionema cordifolium*]**	53.3	YP_001122954.1
	11/*A.t*$_{oe1}$	100	**Large subunit of riblose-1,5-bisphosphate carboxylase/oxygenase (chloroplast) [*Aethionema grandiflorum*]**	53.7	YP_001123038.1
	11/*A.t*$_{oe1}$	100	**Ribulosebisphosphate carboxylase, partial [*Arabidopsis thaliana*]**	47.6	BAA19595.1
	11/*A.t*$_{oe1}$	100	**Ribulose-1,5-bisphosphate carboxylase/oxygenase large subunit, partial (chloroplast) [*Arabis stelleri*]**	47.6	BAA19597.1
	11/*A.t*$_{oe1}$	100	**Ribulose-1,5-bisphosphate carboxylase/oxygenase large subunit, partial (chloroplast) [*Rorippa indica*]**	47.6	BAA19601.1
**QRR**	12/*A.t*$_{oe1}$	100	**Ribulose-1,5-bisphosphate carboxylase/oxygenase large subunit (chloroplast) [*Arabidopsis thaliana*]**	53.0	NP_051067.1
	12/*A.t*$_{oe1}$	100	**Ribulose-1,5-bisphosphate carboxylase/oxygenase large subunit, partial (chloroplast) [*Aethionema saxatile*]**	51.6	AAS88130.1
	12/*A.t*$_{oe1}$	100	**Ribulose-1,5-bisphosphate carboxylase/oxygenase large subunit (chloroplast) [*Olimarabidopsis pumila*]**	53.0	YP_001123123.1
	12/*A.t*$_{oe1}$	100	**Ribulose-1,5-bisphosphate carboxylase/oxygenase large subunit (chloroplast) [*Crucihimalaya wallichii*]**	53.0	YP_001123470.1
	12/*A.t*$_{oe1}$	100	**Large subunit of riblose-1,5-bisphosphate carboxylase/oxygenase (chloroplast) [*Aethionema grandiflorum*]**	53.7	YP_001123038.1
	12/*A.t*$_{oe1}$	100	**Ribulose-1,5-bisphosphate carboxylase/oxygenase large subunit, partial (chloroplast) [*Arabis stelleri*]**	47.6	BAA19597.1
	12/*A.t*$_{oe1}$	100	**Ribulosebisphosphate carboxylase, partial [*Arabidopsis thaliana*]**	47.6	BAA19595.1
	12/*A.t*$_{oe1}$	100	**Ribulose-1,5-bisphosphate carboxylase/oxygenase large subunit, partial (chloroplast) [*Polanisia dodecandra*]**	49.6	AAO38787.1
	12/*A.t*$_{oe1}$	100	**Ribulose-1,5-bisphosphate carboxylase/oxygenase large subunit, partial (chloroplast) [*Chamira circaeoides*]**	50.0	CAJ86803.1

	12/*A.t*$_{oe1}$	100	**Ribulose-1,5-bisphosphate carboxylase/oxygenase large subunit, partial (chloroplast) [*Heliophila pubescens*]**	51.5	CAJ86804.1
**QRR**	13/*A.t*$_{wt}$	100	**Ribulose-1,5-bisphosphate carboxylase/oxygenase large subunit (chloroplast) [*Arabidopsis thaliana*]**	53.0	NP_051067.1
	13/*A.t*$_{wt}$	100	**Ribulose-1,5-bisphosphate carboxylase/oxygenase large subunit (chloroplast) [*Capsella bursa-pastoris*]**	53.0	YP_001123381.1
	13/*A.t*$_{wt}$	100	**Ribulose-1,5-bisphosphate carboxylase/oxygenase large subunit (chloroplast) [*Crucihimalaya wallichii*]**	53.0	YP_001123470.1
	13/*A.t*$_{wt}$	100	**Large subunit of riblose-1,5-bisphosphate carboxylase/oxygenase (chloroplast) [*Draba nemorosa*]**	53.0	YP_001123558.1
	13/*A.t*$_{wt}$	100	**Large subunit of riblose-1,5-bisphosphate carboxylase/oxygenase (chloroplast) [*Aethionema cordifolium*]**	53.3	YP_001122954.1
	13/*A.t*$_{wt}$	100	**Large subunit of riblose-1,5-bisphosphate carboxylase/oxygenase (chloroplast) [*Aethionema grandiflorum*]**	53.7	YP_001123038.1
	13/*A.t*$_{wt}$	100	**Ribulose-1,5-bisphosphate carboxylase/oxygenase large subunit, partial (chloroplast) [*Aethionema saxatile*]**	51.6	AAS88130.1
	13/*A.t*$_{wt}$	100	**Ribulose-1,5-bisphosphate carboxylase/oxygenase large subunit (chloroplast) [*Olimarabidopsis pumila*]**	53.0	YP_001123123.1
	13/*A.t*$_{wt}$	100	**Large subunit of riblose-1,5-bisphosphate carboxylase/oxygenase (chloroplast) [*Arabis hirsuta*]**	53.0	YP_001123207.1
	13/*A.t*$_{wt}$	100	**Ribulosebisphosphate carboxylase, partial [*Arabidopsis thaliana*]**	47.6	BAA19595.1

Figure S1. Clustal Omega (v1.2.4, EMBL-EBI) multiple sequence alignment of Type II metacaspases from *Arabidopsis thaliana* and *Dunaliella salina* showing the conserved catalytic amino acid residues HYSGHGT and CHSG of the catalytic diad.

```
Dusal.0158s00011.1       -------MGRSALLIGCNYPGTKAALQGCVNDVWDFKQTLMECMGFDEDNIAIMVDTDQE
AMC9sp|Q9FYE1|1-325      MDQQGMVKKRLAVLVGCNYPNTRNELHGCINDVLAMKETILSRFGFKQDDIEVLTDEPES
AMC8sp|Q9SA41|1-381      -------MAKKALLIGINYPGTAVELRGCVNDVHRMQKCLIELYGFANKDIVIMIDTDKS
AMC7sp|Q6XPT5|1-403      -------MAKRALLIGINYPGTTEELQGCVNDVHRMHKCLVDRFGFAEEDITVLIDTDES
AMC4sp|O64517|1-418      -------MTKKAVLIGINYPGTKAELRGCVNDVRRMYKCLVERYGFSEENITVLIDTDES
AMC6sp|O64519|1-368      -------MAKKALLIGINYVGTKAELRGCVNDVRRMRISLVERYGFSEENIKMLIDTDSS
AMC5sp|O64518|1-410      -------MAKKAVLIGINYPGTKAELRGCVNDVRRVHKSLVDRFGFSERNITELIDTDES
                                  : *:*:* ** .*   *:**:***  .   ::.  ** : :*  : *  ..

Dusal.0158s00011.1       YPQPTGKNIRAYLNKMVDEA--EDGDLLFLHYSGHGTQLPTDDP--EEPDGKNECICPSD
AMC9sp|Q9FYE1|1-325      KVKPTGANIKAALRRMVDKAQAGSGDILFFHYSGHGTRIPSVKS--AHPFKQDEAIVPCD
AMC8sp|Q9SA41|1-381      CIQPTGKNICDELDNLIASG--QSGDFLVFHYSGHGTRIPPGIEDSEDPTGFDECITPCD
AMC7sp|Q6XPT5|1-403      YTQPTGKNIRQALSELIKPA--KSGDVLFVHYSGHGTRVPPETG-EEDDTGFDECIVPSD
AMC4sp|O64517|1-418      STQPTGKNIRRALADLVESA--DSGDVLVVHYSGHGTRLPAETG-EDDDTGFDECIVPCD
AMC6sp|O64519|1-368      SIKPTGKNIRQALLDLVEPA--KSGDVLFVHYSGHGTRLPAETG-EDDDTGYDECIVPSD
AMC5sp|O64518|1-410      STKPTGKNIRRALLNLVESA--KPGDVLVVHYSGHGTRLPAETG-EDDDTGYDECIVPCD
                           :*** **   *  ::  .    **.*..*******::*       .    :*.* *.*

Dusal.0158s00011.1       LDVISDNDLTKIFAPLSKKNVKLTFVADCCHSGTLLDHKEVIITGPKDNKRPPPQAN--A
AMC9sp|Q9FYE1|1-325      FNLITDVDFRELVNQL-PKGTSFTMISDSCHSGGLIDKEKEQIGPSSVSSNISP------
AMC8sp|Q9SA41|1-381      MNLIKDQQFREMVSRV-KEGCQLTIISDSCHSGGLIQEVKEQIGESHMKPVDKVKEQIEE
AMC7sp|Q6XPT5|1-403      LNPIPDDDFRDLVEQV-PEGCQITIVSDSCHSGGLIDEAKEQIGESTTTKPNRESKV---
AMC4sp|O64517|1-418      MNLITDDDFRDLVDKV-PPGCRMTIISDSCHSGGLIDEAKEQIGESTKKEAEDEDES---
AMC6sp|O64519|1-368      MNLITDDDFRDLVDMV-PKDCPITIISDSCHSGGLIDEAKEQIGESTKKKKD--------
AMC5sp|O64518|1-410      MNLITDDEFRDLVEKV-PKEAHITIISDSCHSGGLIDEAKEQIGESTKKKPKKE------
                         :: * * :: .:.  :      :*:::*.**** *::. :  *     .

Dusal.0158s00011.1       RSME---------------A-----------------------LQAALGAGDDSRDIQIR
AMC9sp|Q9FYE1|1-325      ------------------------------------------------AIETTNKTITSR
AMC8sp|Q9SA41|1-381      SHMKQPKLGIASYFLNIVMNLLATCGVSKSQR--DRGGGEESFRGEIELEKDETLDIKTR
AMC7sp|Q6XPT5|1-403      ---SSFEFEFKNCLHSIFVKLLAFCGIGSSH----------VETREIVEVGEGDEVVRSR
AMC4sp|O64517|1-418      -EESSSRFGFRKFLRSKVEGAIESRGFHIGGNKKDEDEAEEIETKEIELEDGETIHAKDK
AMC6sp|O64519|1-368      -SGDSSTIN--------------------------------------KETEAEIIEVGNR
AMC5sp|O64518|1-410      -SGGSSGLGIKGFVREAVEEALESKGIAIPHHKDEK---DENKTKELKLEDGAKVHVVNK
                                                                                    :

Dusal.0158s00011.1       SLPLEVYKELLSGKLGG--VDFGDLPVSLIKGFGDESMERWARLFPVIADLQKRGQDTSK
AMC9sp|Q9FYE1|1-325      ALPFKAVLDHLSSLTGITT---SDIGTHLLELFGRDAGLKFRLPAMDLMDLLE-------
AMC8sp|Q9SA41|1-381      YLPFESYLSLLKEQTGQTNIEPVRIRQTLLKLFGEDPSPNRQRGLSDLG------N----
AMC7sp|Q6XPT5|1-403      YLPLERFIELLKQQTGQDNIEIGKIRPTLFDVFGEDSSPKIKKFMKVILTKLRKTND---
AMC4sp|O64517|1-418      SLPLQTLIDILKQQTGNDNIEVGKIRPSLFDAFGDDSSPKVKKFMKVILGKLQAGNG---
AMC6sp|O64519|1-368      SLPLETLIDMLKQETGNDDIEVGKIRTTLFDMFGDDSSPKVKKFMNVILSNLQETTTTIQ
AMC5sp|O64518|1-410      SLPLQTLIDILKQNTGNNDIEVGKIRPTLFNVFGEDASPKVKKFMKVILTKLQEGKT---
                          **::   . *.   *        :

Dusal.0158s00011.1       T-----LNTLRESA---------------------------ETLVGL-KKAREPLNENVG
AMC9sp|Q9FYE1|1-325      ------------------------------------------------TMTAREKHVDSG
AMC8sp|Q9SA41|1-381      -----------------------------------------CEVDAGDSGASRLNAVTDNG
AMC7sp|Q6XPT5|1-403      --QSTLLGKIEESARGYIEETLND-EHYMKPAMQAQVKSDREIYGG--RSSNG-LFPDRG
AMC4sp|O64517|1-418      -EEGGLMGMLGKLASGFLEGKLND-EDYVKPAMQTHVGSKEEVYAG--GSRGSVPLPDSG
AMC6sp|O64519|1-368      TVSDEVLGSVENLAQEFLEQKLSDDVK---P-------AIQDVYAG--AINGA--LPDNG
AMC5sp|O64518|1-410      --EGGILGMIGKLAQEFLKHKLNDDEEYVKPAMKTHVGNKQEVYAG--ASNGS--LADNG

Dusal.0158s00011.1       ILLSGCLERECAADVRRK--NSVPHGAFSHAVNMLLRQMHKDGIIATNRSVNLGVRESLS
AMC9sp|Q9FYE1|1-325      ILMSGCQADETSADVGVG--NGKAYGAFSNAIQRVLNEN---EGAMKNKQLVMMARDVLE
AMC8sp|Q9SA41|1-381      ILLSGCQTDQRSEDVYVT-RTGKAYGAFSDAIQMILSAPRKDKKKITNKELVSEARVFLK
AMC7sp|Q6XPT5|1-403      ILLSGCQTDETSADVKKKG---EAFGAFSNAIQMVLSET-DHKDKITNKEMVLRAREILK
AMC4sp|O64517|1-418      ILISGCQTDQTSADATPAGKPTEAYGAMSNSIQTILEET-D--GEISNREMVTRARKALK
AMC6sp|O64519|1-368      ILISGCQTDQTSSDASPPGHPELAYGALTNAIQIIIGET-K--GKISNKDLVLKARKLLR
AMC5sp|O64518|1-410      ILISGCQTDQTSADASPQGHPEMAYGAFTNAVQIILEET-K--GMITYKELVLKARKLLK

Dusal.0158s00011.1       KSGLKQSPCLEGSEKWADAAFVLCDEEGSI*
AMC9sp|Q9FYE1|1-325      RLGFHQHPCLYCSDQNADATFLSQP------
AMC8sp|Q9SA41|1-381      KRGYSQRPGLYCHDRFVDKPFICY-------
AMC7sp|Q6XPT5|1-403      KQMFIQRPGLYCNDRFVNAPFIC--------
AMC4sp|O64517|1-418      KQGFTQQPGLYCHDGYANAPFIC--------
AMC6sp|O64519|1-368      KQGFDQRPGLYCNDAYVNARFIC--------
AMC5sp|O64518|1-410      KQGFSQRPGLYCSDSFVNAPFIC--------
```
